# Supplementary material for: Tranilast directly targets NLRP3 to treat inflammasome‐driven diseases
Source: EMBO Mol Med. 2018 Mar 12;10(4):e8689. doi: 10.15252/emmm.201708689 (PMC5887903; doi:10.15252/emmm.201708689)
Supplement: Supplementary file 1 — Appendix [file EMMM-10-e8689-s001.pdf]

Appendix

Table of contents **page**

Appendix Figure S1	2
Appendix Figure S2	3
Appendix Figure S3	4
Appendix Figure S4	5
Appendix Figure S5	6
Appendix Figure S6	7
Appendix Figure S7	8
Appendix Figure S8	9
Appendix Table S1	10

Appendix Figure S1

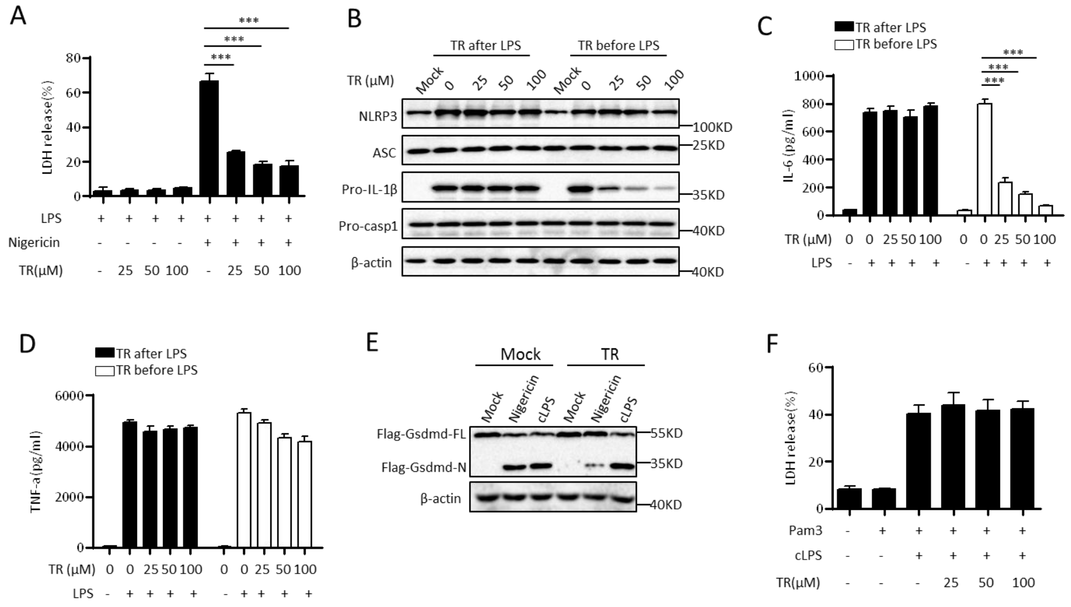

Appendix Fig. S1. Role of TR in NLRP3 inflammasome activation. (A) Assay for LDH release in the culture supernatants of LPS-primed BMDMs treated with different doses of TR for 30 min and then left stimulated with nigericin for 1 h. **(B)** Immunoblot analysis of the indicated proteins in lysates from BMDMs treated with LPS for 3 h and left stimulated with different doses of TR for 30 min (TR after LPS), or BMDMs treated with different doses of TR for 30 min and then stimulated with LPS for 3 h (TR before LPS). **(C, D)** ELISA of IL-6 or TNF-α in supernatants from BMDMs described in **(B)**. **(E)** Flag-Gsdmd reconstituted Gsdmd$^{-/-}$ iBMDMs cells were treated with LPS (50 ng/ml) or Pam3 (400 ng/ml) for 3 h. After that, the cells were incubated with TR (100 μM) for 30 min and then stimulated with nigericin (6 μM) for 1 h or cLPS (1 μg/mL) for 20 h. The cell lysates were immunoblotted for analysis of the Gsdmd cleavage. **(F)** Assay for LDH release in the culture supernatants of Pam3-primed BMDMs treated with different doses of TR for 30 min and then left stimulated with cLPS for 18 h. Data are from three independent experiments with biological duplicates in each **(A, C, D, F)**; mean and s.e.m of n = 6) or are representative of three independent experiments **(B, E)**. Statistics were analyzed using an unpaired Student’s t test: ***P <0.001.

Source data are available online for this figure.

Appendix Figure S2

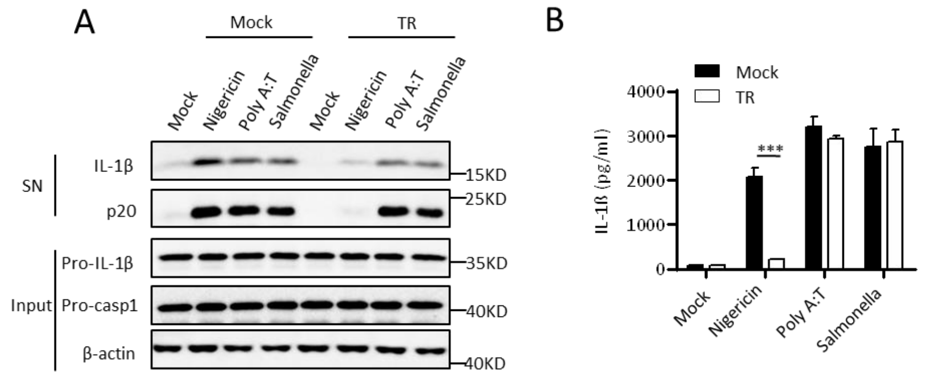

Appendix Fig. S2. Role of TR in AIM2 or NLRC4 inflammasome activation. (A, B) Immunoblot analysis of IL-1β and cleaved caspase-1 (p20) **(A)** or ELISA of IL-1β **(B)** in culture supernatants of LPS-primed BMDMs treated with of TR (100 μM) and then stimulated with nigericin for 30min, cytosolic poly A:T or Salmonella for 4h. Data are from three independent experiments with biological duplicates in each (**B**; mean and s.e.m. of $n = 6$) or are representative of at least three independent experiments **(A)**. Statistics were analyzed using an unpaired Student's t test: ***P < 0.001.

Source data are available online for this figure.

Appendix Figure S3

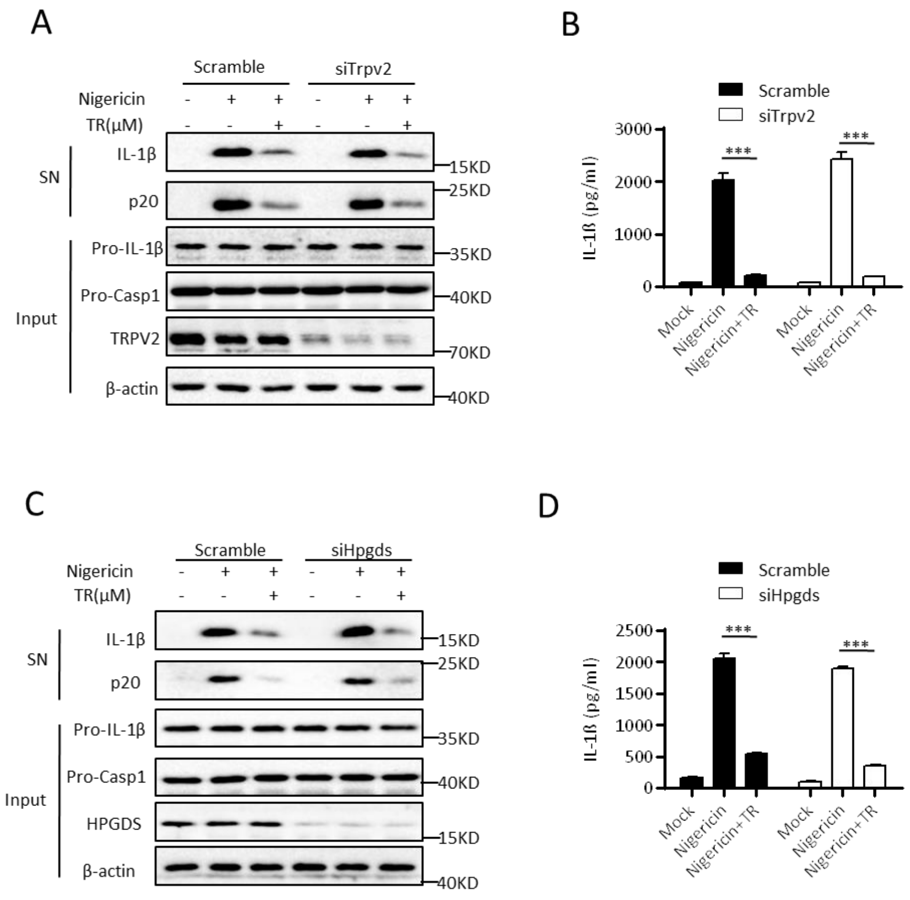

Appendix Fig. S3. The inhibitory effects of TR on NLRP3 inflammasome activation are independent of TRPV2 or HPGDS. (A, C) Immunoblot analysis of IL-1β and cleaved caspase-1 (p20) in culture supernatants of LPS-primed BMDMs transfected with siRNA against Tprv2 **(A)** or Hpgds **(C)** and left stimulated with nigericin for 30min. **(B, D)** ELISA of IL-1β in the culture supernatants described in **(A)** or **(C)**. Data are from three independent experiments with biological duplicates in each (**B, D)**; mean and s.e.m of n = 6) or are representative of three independent experiments **(A, C)**. Statistics were analyzed using an unpaired Student's t test: ***P <0.001.

Source data are available online for this figure.

Appendix Figure S4

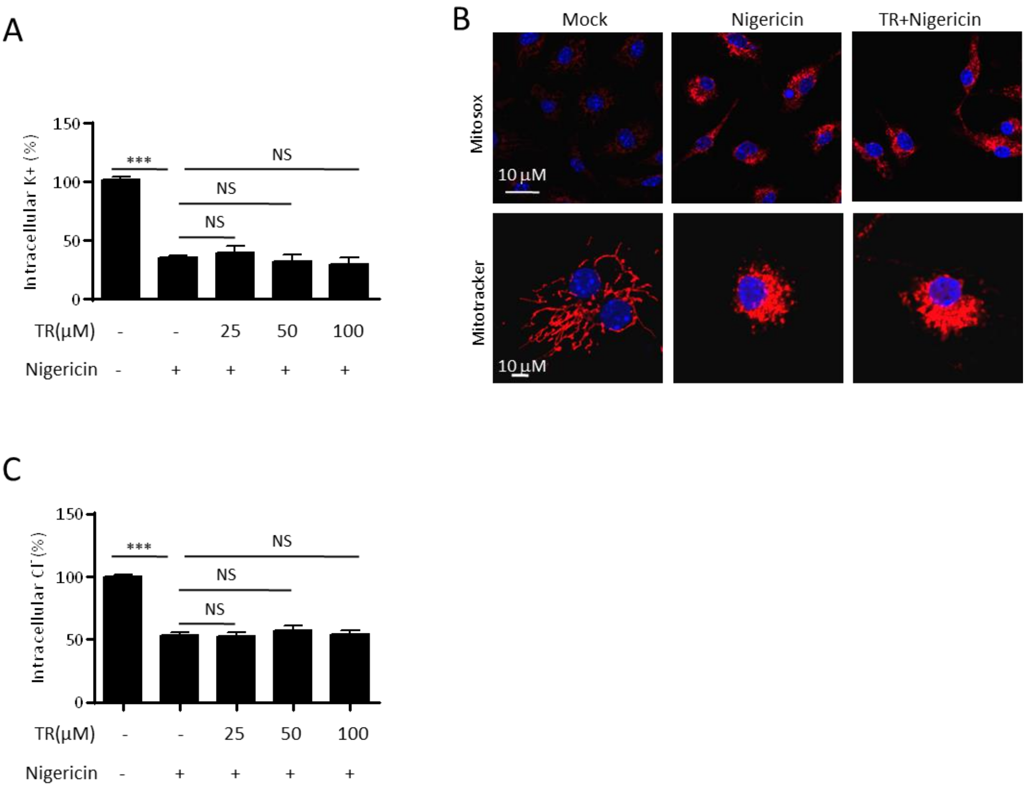

Appendix Fig. S4. TR has no effects on potassium efflux, mitochondrial damage or chloride efflux. (A) Qualification of potassium efflux in LPS-primed BMDMs treated with different doses of TR and then left stimulated with nigericin for 30 min. **(B)** Confocal microscopy analysis in LPS-primed BMDMs with TR and then left stimulated with nigericin for 30 min, followed by staining with Mitosox, Mitotracker red and DAPI. **(C)** Qualification of chloride efflux in LPS-primed BMDMs treated with different doses of TR and then left stimulated with nigericin for 15 min. Data are from three independent experiments with biological duplicates in each **(A, C)**; mean and s.e.m of n = 6) or are representative of three independent experiments **(B)**. Statistics were analyzed using an unpaired Student's t test: ***P <0.001, NS, not significant.

Appendix Figure S5

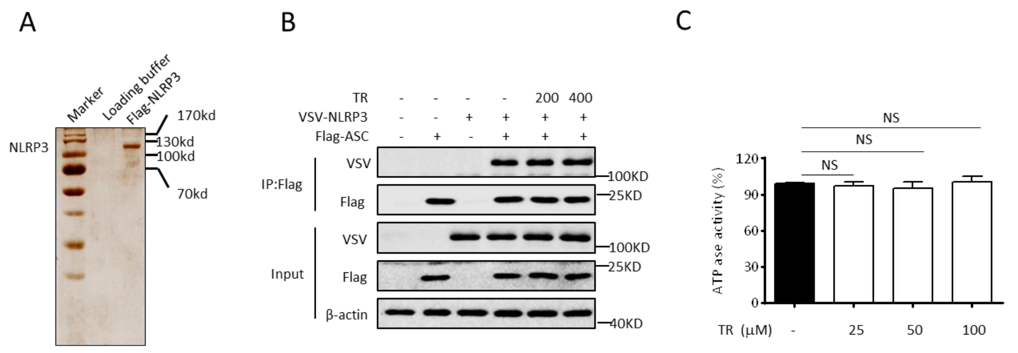

Appendix Fig. S5. TR has no effects on direct NLRP3-ASC interaction and ATPase activity of NLRP3. (A) silver stainning of recombinant human NLRP3 protein. **(B)** Immunoprecipitation (IP) and immunoblot analysis of the interaction of Flag-ASC and VSV-NLRP3 in the lysates of HEK-293T cells. TR was added at 8 h post-transfection. **(C)** *In vitro* ATPase assay of purified NLRP3 proteins in the presence of different doses of TR. Data are from three independent experiments with biological duplicates in each **(C)**; mean and s.e.m of n = 6) or are representative of three independent experiments **(B)**. Statistics were analyzed using an unpaired Student's t test: NS, not significant.

Source data are available online for this figure.

Appendix Figure S6

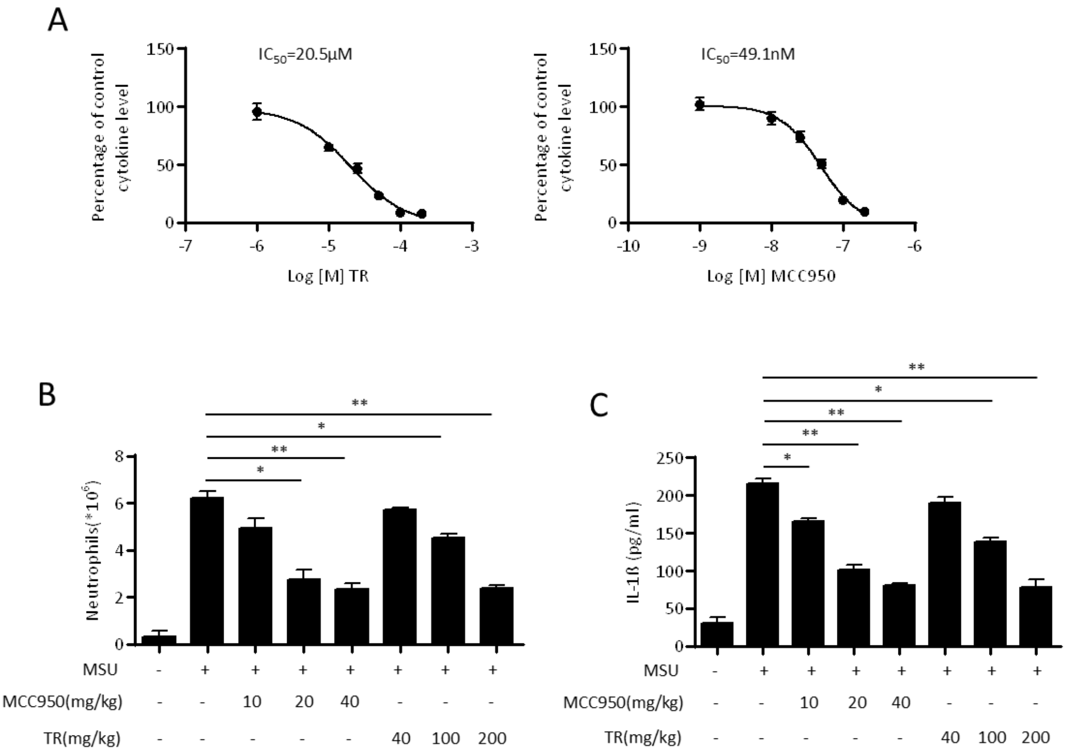

Appendix Fig. S6. Comparison of the activity of TR with MCC950. (A) BMDMs were primed with LPS for 3 hours and then treated with different doses of TR or MCC950 for 30 min and then left stimulated with MSU for another 4 h. Production of IL-1β were measured by ELISA and then the Cytokine level is normalized to that of DMSO-treated control cells. Nonlinear regression analysis was performed, and the curve of Log [M] TR or MCC950 versus the normalized response is presented. Data are from three independent experiments with biological duplicates in each. **(B, C)** FACS analysis of neutrophil numbers **(B)** or ELISA **(C)** of IL-1β in the peritoneal cavity of 10-Week-old male C57BL/6J mice intraperitoneally injected with MSU (1 mg/mouse) with the presence of different doses of TR or MCC950. *n* = 3 mice per group. Statistics were analyzed using an unpaired Student's t test: *P<0.05, **P <0.01.

Appendix Figure S7

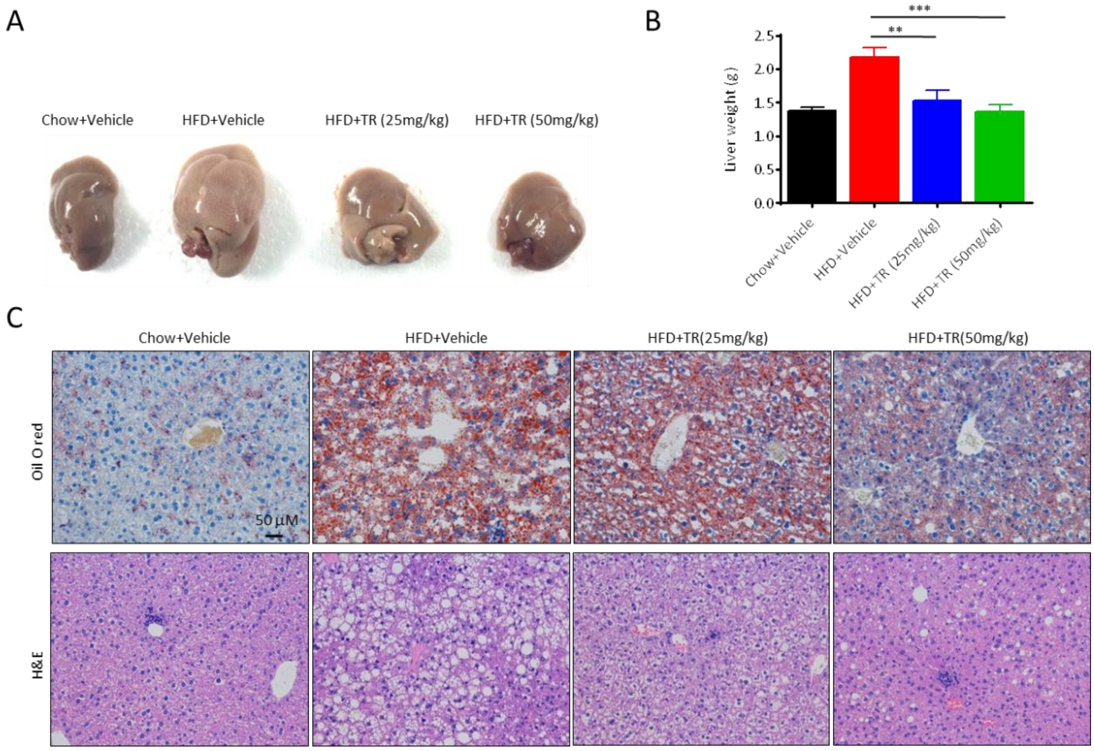

Appendix Fig. S7. The preventive role of TR in HFD-induced hepatic steatosis. (A-C) Representative liver morphology **(A)**, weights of the whole livers **(B)** representative Oil red O or H&E staining of liver sections **(C)** of C57BL/6J mice at week 12 after initiation of HFD with or without oral TR treatment. n = 7 per group. Data are shown as mean and s.e.m. and are representative of two independent experiments. Statistics were analyzed using an unpaired Student's t test: **P <0.01, ***P <0.001.

Appendix Figure S8

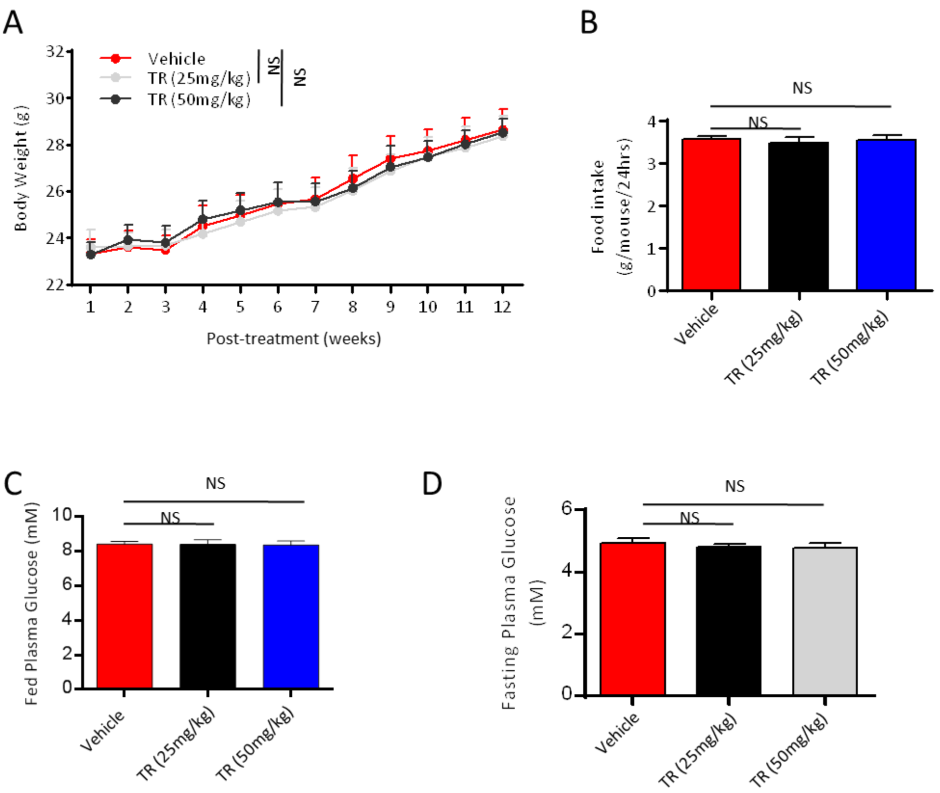

Appendix Fig. S8. The role of TR in normal diet-fed mice. (A) Body weights measured at the indicated time points after 6-Week-old male C57BL/6J mice fed with normal diet with or without oral TR. n = 12 per group. **(B)** Daily food intake of the male C57BL/6J mice fed with normal diet with or without oral TR treatment. n = 6 per group. **(C, D)** Fed **(C)** or fasting **(D)** blood glucose concentrations of male C57BL/6J mice after fed with normal diet for 12 weeks with indicated dose of oral TR. n = 6 per group. Data are shown as mean and s.e.m. and are representative of two independent experiments. Statistics were analyzed using an unpaired Student's t test: NS, not significant.

Appendix Table S1. All p values for figures and appendix figures.

Figures		P values
Figure 1B	0 μ M vs 25 μ M	9.57E-05
	0 μ M vs 50 μ M	7.04219E-07
	0 μ M vs 50 μ M	3.91961E-08
Figure 1G	Mock vs TR (MSU)	1.35895E-08
	Mock vs TR (Nigericin)	5.09207E-13
	Mock vs TR (ATP)	1.16355E-08
	Mock vs TR (Alum)	9.47532E-06
Figure 1I	0 μ M vs 25 μ M	0.003682
	0 μ M vs 50 μ M	0.000110112
	0 μ M vs 50 μ M	1.24126E-05
Figure 4A	MSU vs MSU+TR (Neutrophils)	0.0359
Figure 4B	MSU vs MSU+TR (IL-1 β)	6.47213E-06
Figure 4C	MSU vs MSU+TR (WT,1h)	0.000582467
	MSU vs MSU+TR (WT,6h)	0.000270749
	MSU vs MSU+TR (WT,12h)	2.722E-05
	MSU vs MSU+TR (WT,24h)	6.38649E-05
Figure 4D	Mock vs MSU (WT)	1.04026E-05
	MSU vs MSU+TR (WT)	5.16447E-05
	MSU vs MSU+TR ($NLRP3^{-/-}$)	0.281989785
Figure 4F	Vehicle vs TR (WT)	0.725637186
	Vehicle vs TR ($NLRP3^{-/-}$)	3.11194E-05
Figure 4G	Vehicle vs TR	0.0002
Figure 5A	Vehicle vs 25mg/kg	1.02717E-05
	Vehicle vs 50mg/kg	4.95289E-11
Figure 5B	Vehicle vs 25mg/kg	0.32563
	Vehicle vs 50mg/kg	0.107683
Figure 5C	Vehicle vs 25mg/kg (5 week)	0.00117114
	Vehicle vs 50mg/kg (5 week)	6.04219E-05
	Vehicle vs 25mg/kg (7 week)	0.000151838

	Vehicle vs 50mg/kg (7week)	1.17749E-05
	Vehicle vs 25mg/kg (12 week)	2.97502E-05
	Vehicle vs 50mg/kg (12 week)	7.64486E-06
Figure 5D	Vehicle vs 50mg/kg (15min)	0.013237
	Vehicle vs 50mg/kg (30min)	0.004912
	Vehicle vs 50mg/kg (60min)	0.000188
	Vehicle vs 50mg/kg (90min)	0.001197
	Vehicle vs 50mg/kg (120min)	0.02686
Figure 5E	Vehicle vs 50mg/kg (0min)	4.9E-05
	Vehicle vs 50mg/kg (30min)	0.001372
	Vehicle vs 50mg/kg (60min)	0.000154
	Vehicle vs 50mg/kg (90min)	0.000646
	Vehicle vs 50mg/kg (120min)	0.00038
Figure 5F	Vehicle vs 25mg/kg	0.000753243
	Vehicle vs 50mg/kg	0.000658
Figure 5G	Vehicle vs 25mg/kg (WAT,IL-1β)	0.004774307
	Vehicle vs 50mg/kg (WAT,IL-1β)	0.001448
	Vehicle vs 25mg/kg (Liver,IL-1β)	0.005643117
	Vehicle vs 50mg/kg (Liver,IL-1β)	0.001121
Figure 5I	Vehicle vs 25mg/kg (WAT,TNF-α)	0.003158567
	Vehicle vs 50mg/kg (WAT, TNF-α)	0.000538
	Vehicle vs 25mg/kg (Liver, TNF-α)	0.124138192
	Vehicle vs 50mg/kg (Liver, TNF-α)	0.007075
Figure 6A	Vehicle vs TR (WT)	7.2924E-08
	Vehicle vs TR (NLRP3$^{-/-}$)	0.46517267
Figure 6B	Chow vs HFD (WT)	3.47957E-07
	Vehicle vs 50mg/kg (WT)	0.000121359
	Chow vs HFD (NLRP3$^{-/-}$)	1.95429E-05
	Vehicle vs 50mg/kg (NLRP3$^{-/-}$)	0.213422975
Figure 6C	Chow vs HFD (WT)	4.74557E-06
	Vehicle vs 50mg/kg (WT)	0.000997881
	Chow vs HFD (NLRP3$^{-/-}$)	0.007647254
	Vehicle vs 50mg/kg (NLRP3$^{-/-}$)	0.567295689
Figure 6D	Vehicle vs 50mg/kg (15min)	0.011514

	Vehicle vs 50mg/kg (30min)	0.005109
	Vehicle vs 50mg/kg (60min)	0.000171
	Vehicle vs 50mg/kg (90min)	0.025935
	Vehicle vs 50mg/kg (120min)	0.006189
Figure 6E	Vehicle vs 50mg/kg (0in)	0.000233453
	Vehicle vs 50mg/kg (15min)	3.54825E-05
	Vehicle vs 50mg/kg (30min)	8.7594E-05
	Vehicle vs 50mg/kg (60min)	8.85416E-06
	Vehicle vs 50mg/kg (90min)	2.02122E-06
	Vehicle vs 50mg/kg (120min)	0.000760034
Figure 7B	0 μ M vs 50 μ M	0.0005383
	0 μ M vs 100 μ M	2.85273E-05
Figure S1A	0 μ M vs 25 μ M	4.39109E-06
	0 μ M vs 50 μ M	1.60277E-06
	0 μ M vs 100 μ M	5.48235E-06
Figure S1C	0 μ M vs 25 μ M	5.4838E-09
	0 μ M vs 50 μ M	8.20774E-11
	0 μ M vs 100 μ M	1.01455E-13
Figure S2B	Mock vs TR (Nigericin)	7.1145E-05
Figure S3B	Nigericin vs Nigericin+TR (Scramble)	7.71438E-06
	Nigericin vs Nigericin+TR (SiTrpv2)	1.77468E-05
Figure S3D	Nigericin vs Nigericin+TR (Scramble)	1.06478E-08
	Nigericin vs Nigericin+TR (SiHpgds)	4.91146E-12
Figure S4A	Mock vs Nigericin	1.46732E-11
	0 μ M vs 25 μ M	0.49041181
	0 μ M vs 50 μ M	0.845709999
	0 μ M vs 100 μ M	0.65874
Figure S4C	Mock vs Nigericin	1.0959E-05
	0 μ M vs 25 μ M	0.96370117
	0 μ M vs 50 μ M	0.10051408
	0 μ M vs 100 μ M	0.618259
Figure S5C	0 μ M vs 25 μ M	0.545621823
	0 μ M vs 50 μ M	0.465381624

	0 μ M vs 100 μ M	0.837933414
Figure S6B	0mg/kg vs 20mg/kg (MCC950)	0.019150976
	0mg/kg vs 40mg/kg (MCC950)	0.008661686
	0mg/kg vs 100mg/kg (TR)	0.034106192
	0mg/kg vs 200mg/kg (TR)	0.006313
Figure S6C	0mg/kg vs 10mg/kg (MCC950)	0.022879058
	0mg/kg vs 20mg/kg (MCC950)	0.006202435
	0mg/kg vs 40mg/kg (MCC950)	0.002764875
	0mg/kg vs 100mg/kg (TR)	0.012063625
	0mg/kg vs 200mg/kg (TR)	0.007889
Figure S7B	Vehicle vs 25mg/kg	0.007255397
	Vehicle vs 50mg/kg	0.000582478
Figure S8A	Vehicle vs 25mg/kg	0.623043625
	Vehicle vs 50mg/kg	0.963084
Figure S8B	Vehicle vs 25mg/kg	0.610312
	Vehicle vs 50mg/kg	0.946578879
Figure S8C	Vehicle vs 25mg/kg	0.957326
	Vehicle vs 50mg/kg	0.859751963
Figure S8D	Vehicle vs 25mg/kg	0.526191
	Vehicle vs 50mg/kg	0.509358844
