# Supplementary figures and images for: Tranilast directly targets NLRP3 to treat inflammasome‐driven diseases
Source: EMBO Mol Med. 2018 Mar 12;10(4):e8689. doi: 10.15252/emmm.201708689 (PMC5887903; doi:10.15252/emmm.201708689)

Figure S5A

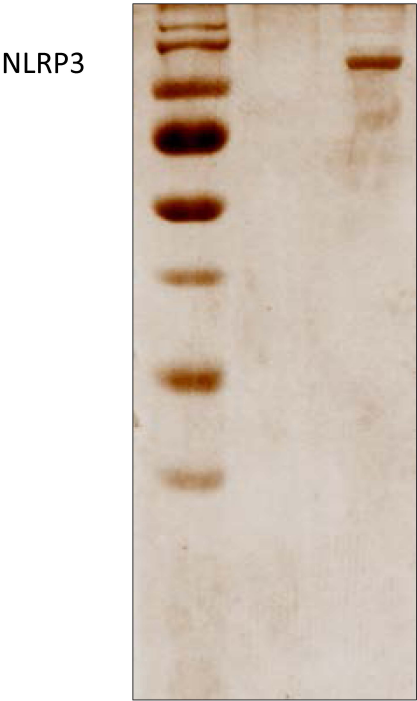

Figure S5B

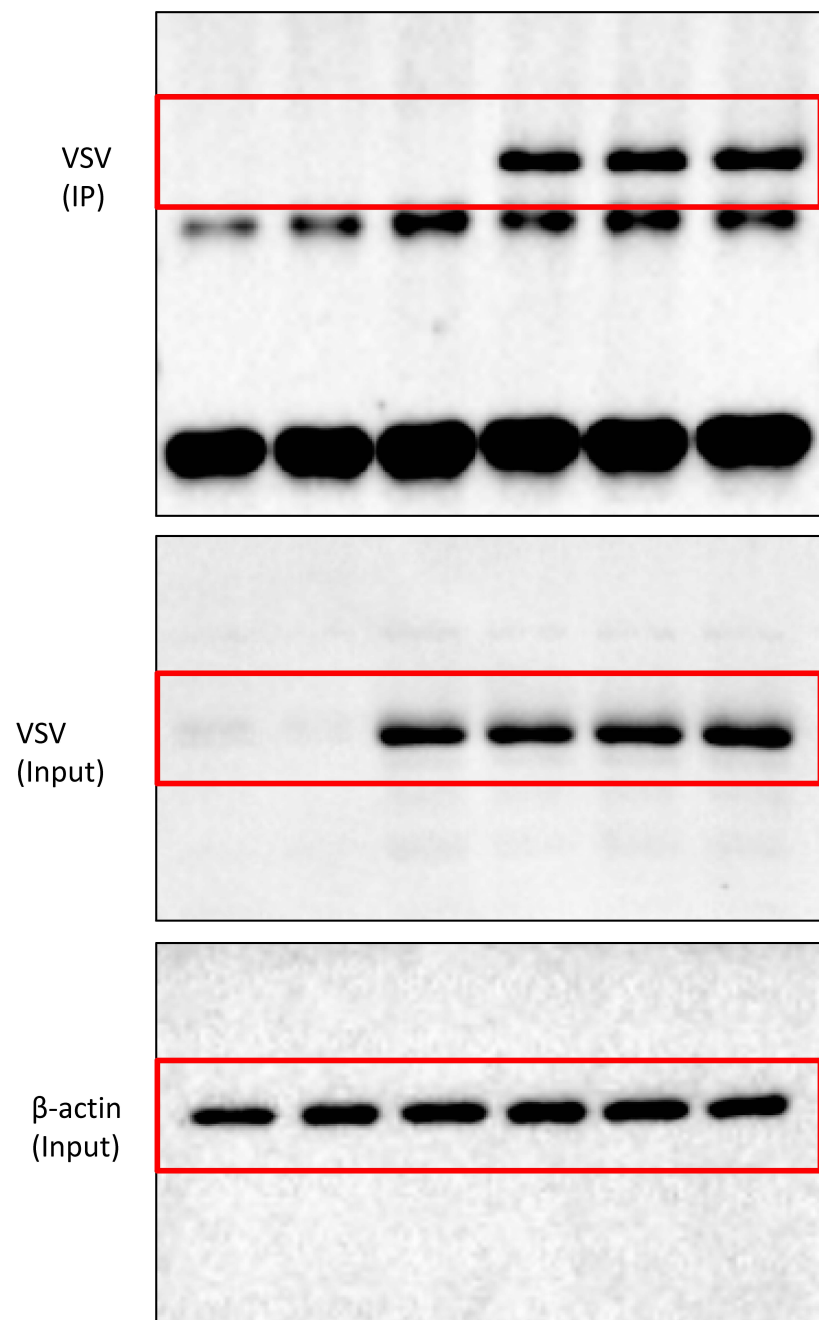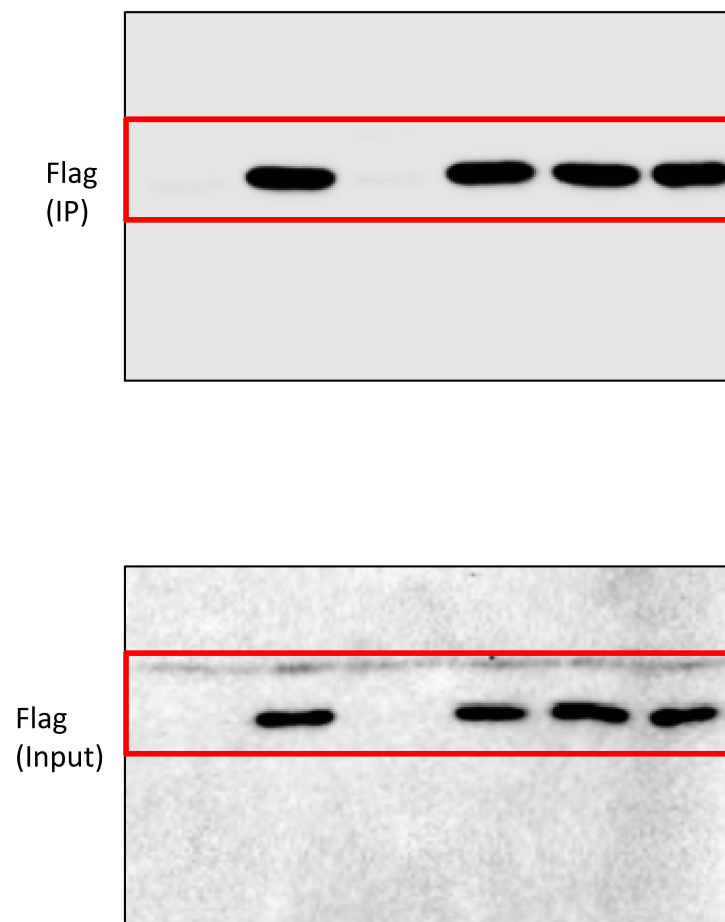

Supplement: Supplementary file 2 — Source Data for Appendix [file EMMM-10-e8689-s007.zip › Figure_S5.pdf]

Figure S1B

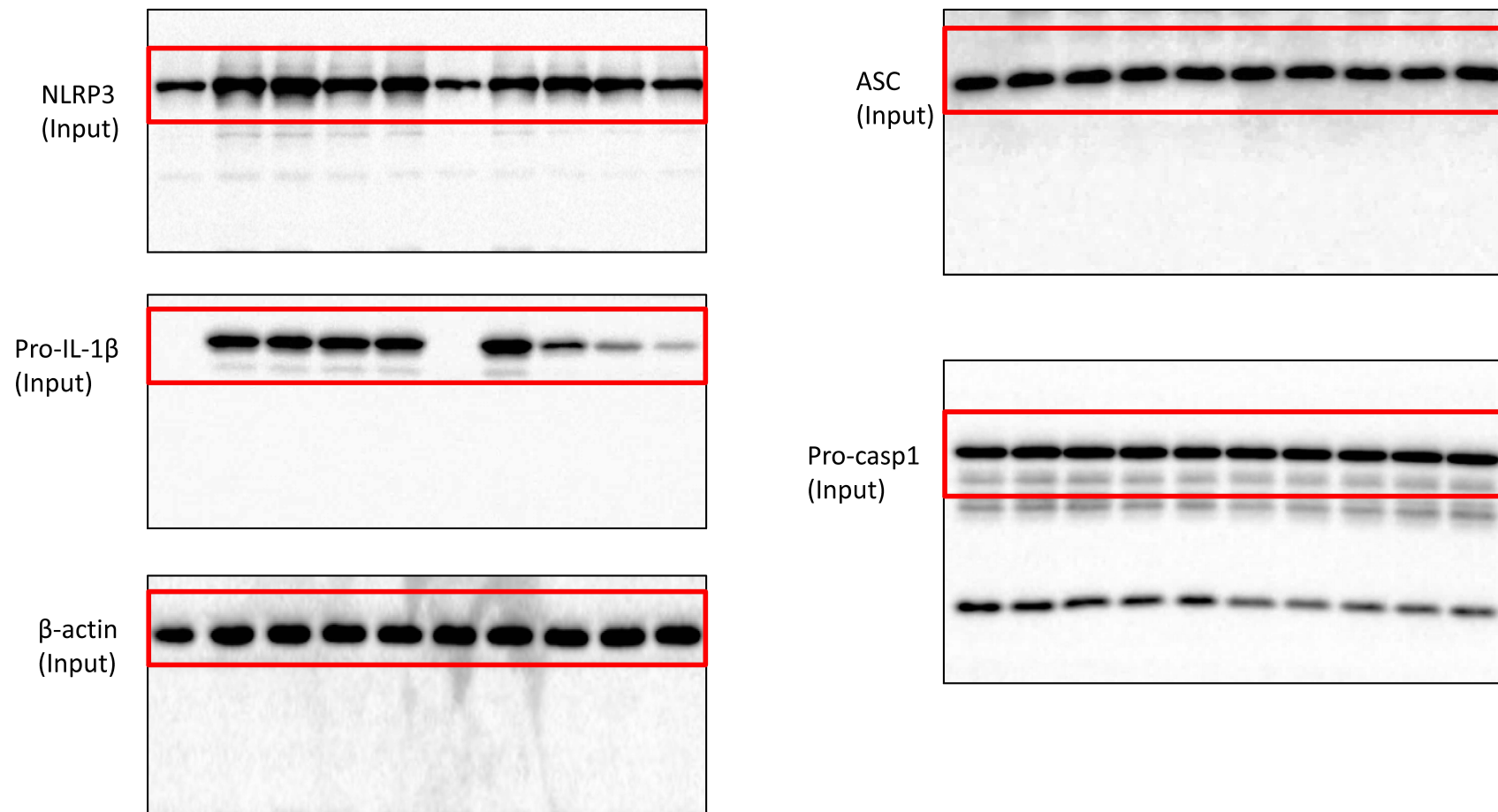

S1E

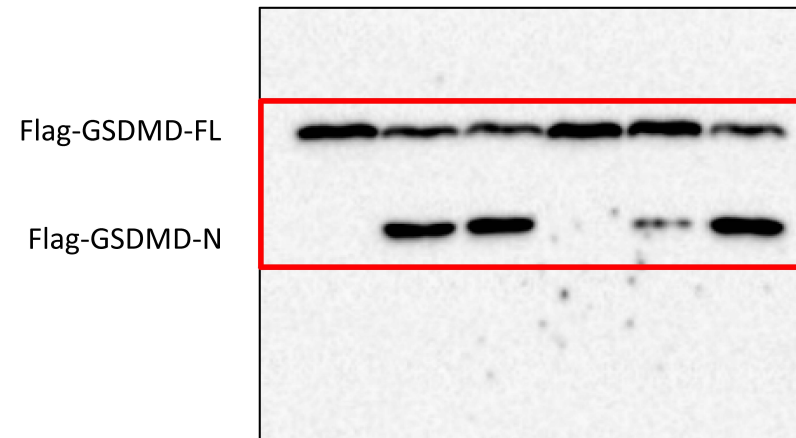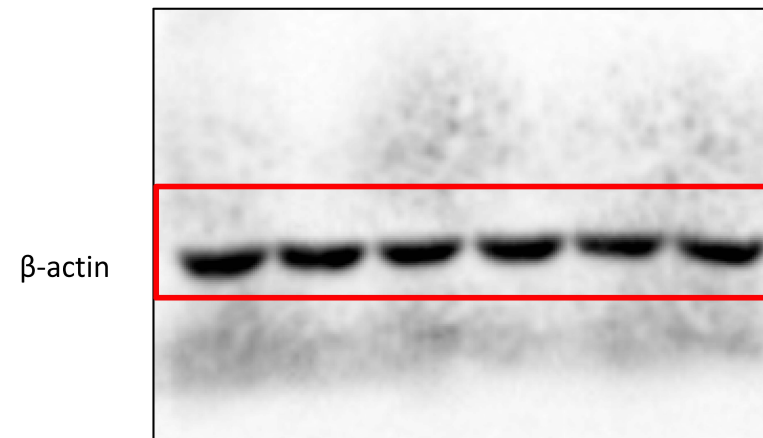

Supplement: Supplementary file 2 — Source Data for Appendix [file EMMM-10-e8689-s007.zip › Figure_S1.pdf]

Figure S2A

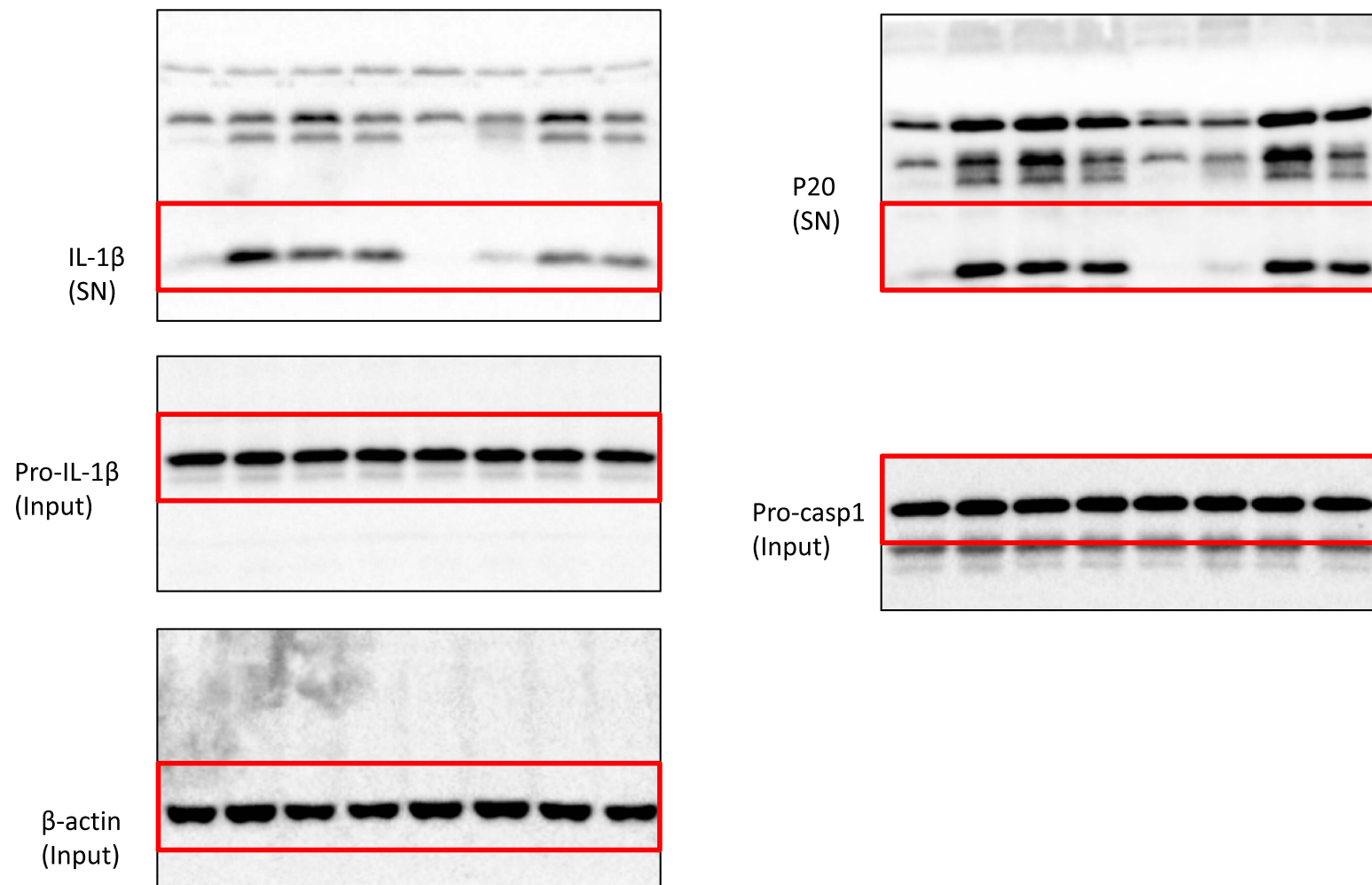

Supplement: Supplementary file 2 — Source Data for Appendix [file EMMM-10-e8689-s007.zip › Figure_S2.pdf]

Figure S3A

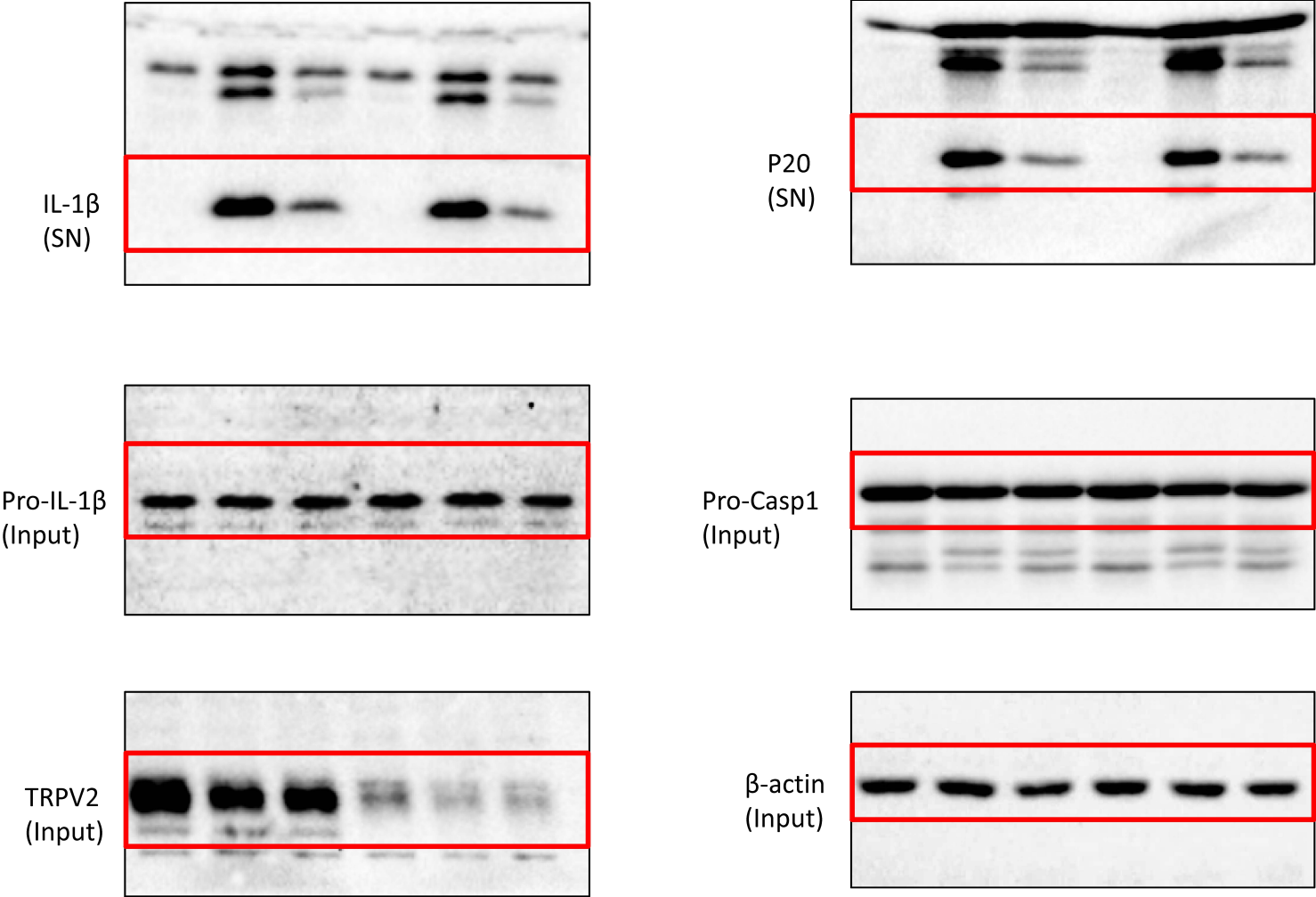

Figure S3C

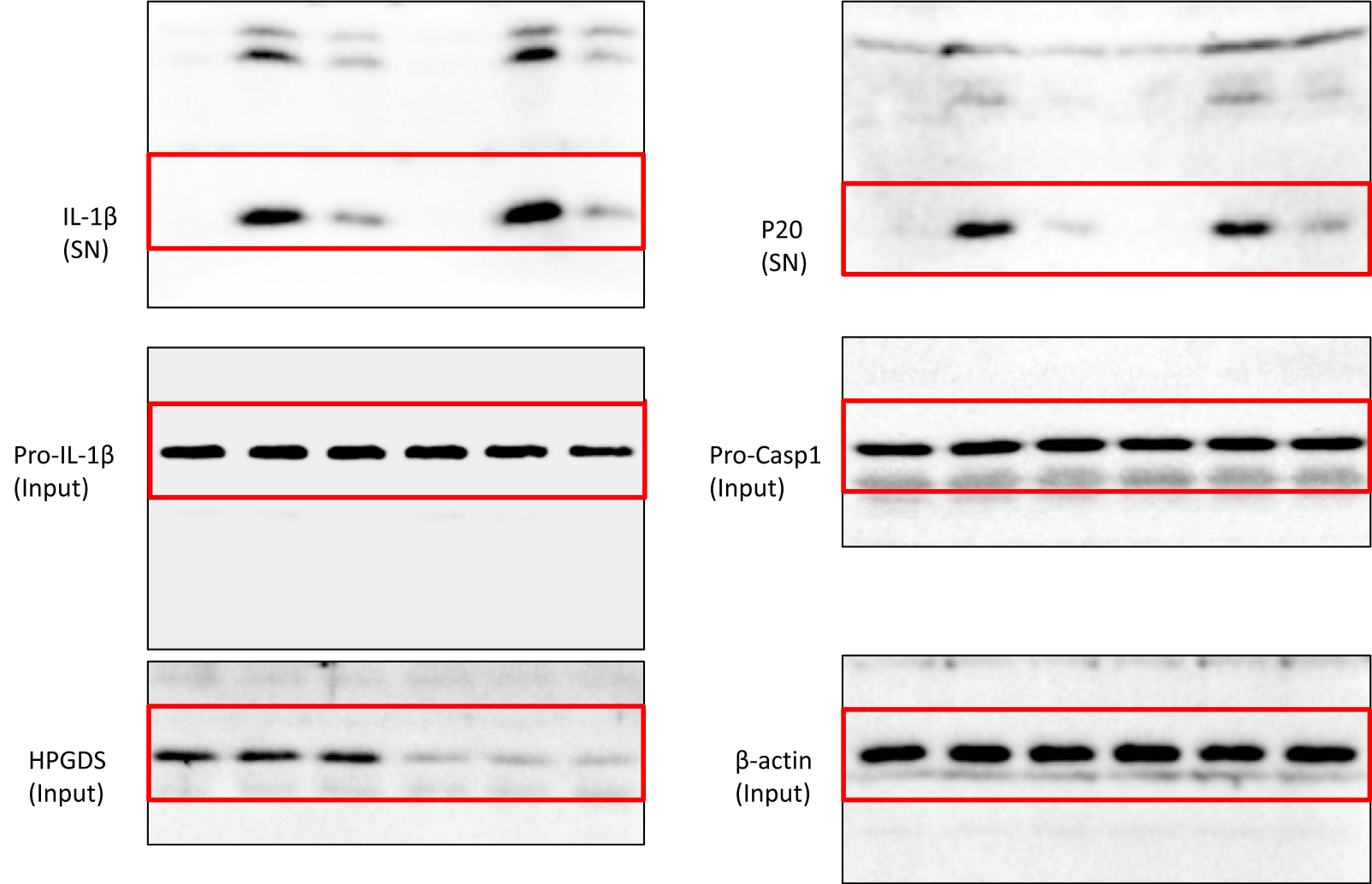

Supplement: Supplementary file 2 — Source Data for Appendix [file EMMM-10-e8689-s007.zip › Figure_S3.pdf]

Figure 1A

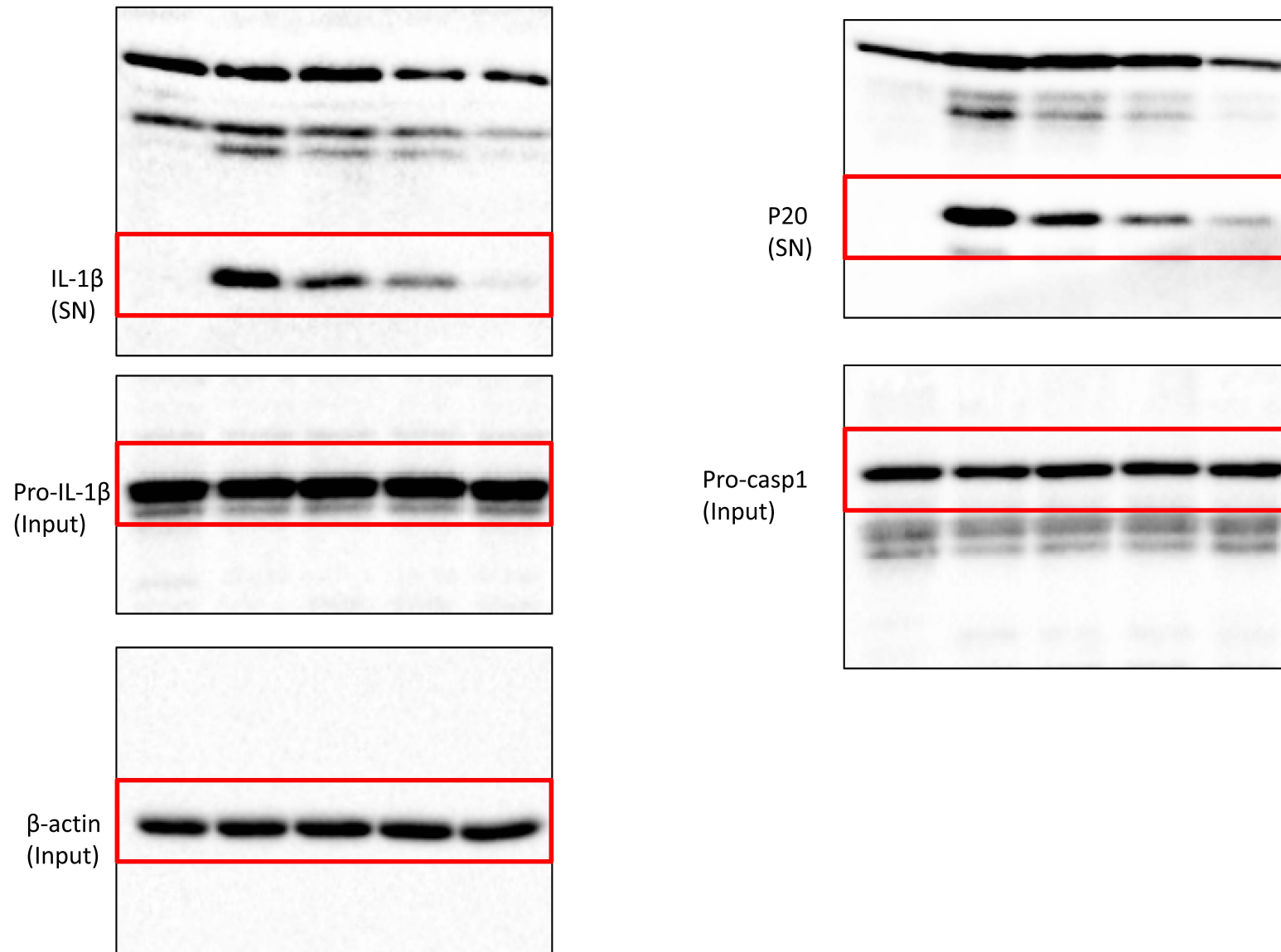

Figure 1E

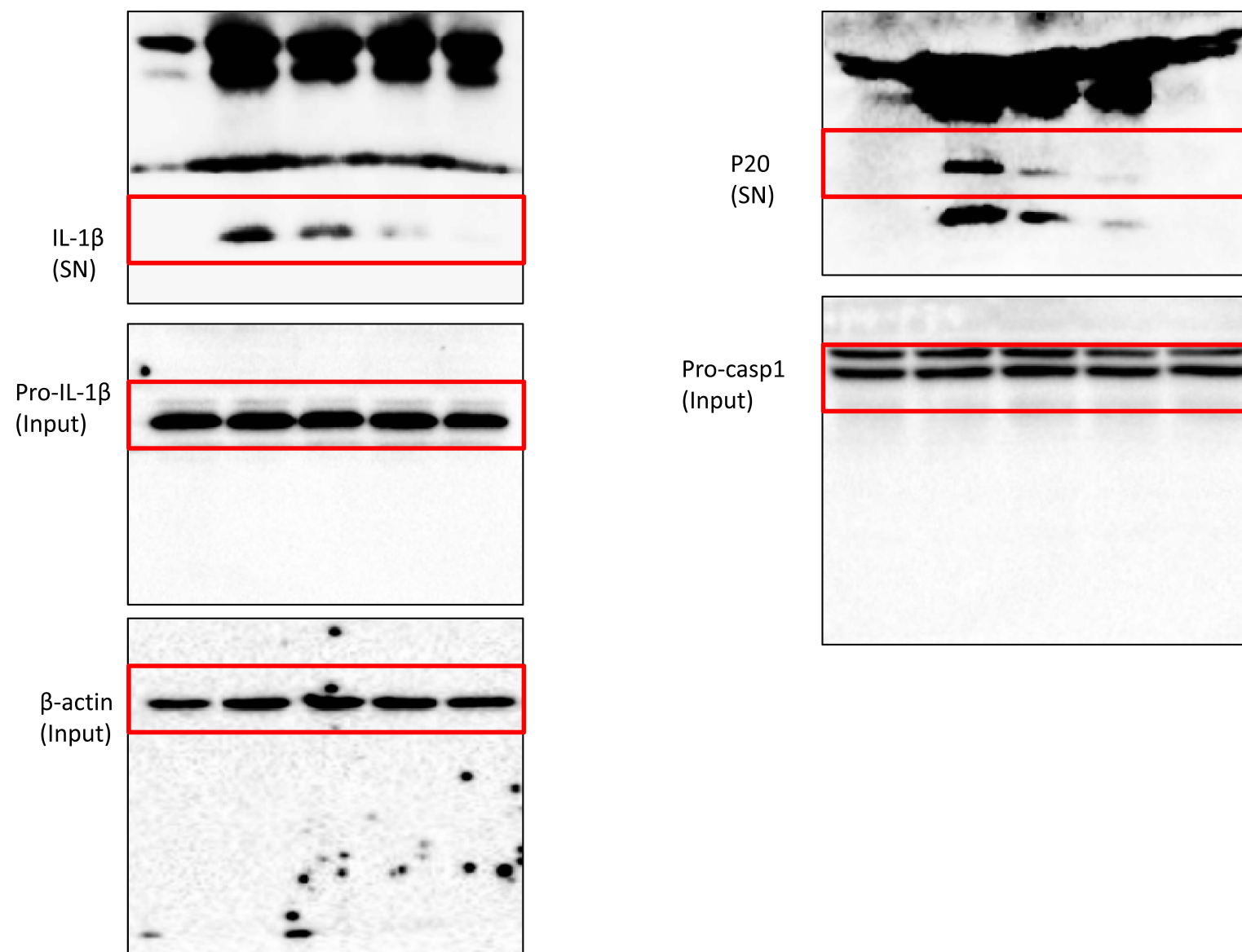

Figure 1F

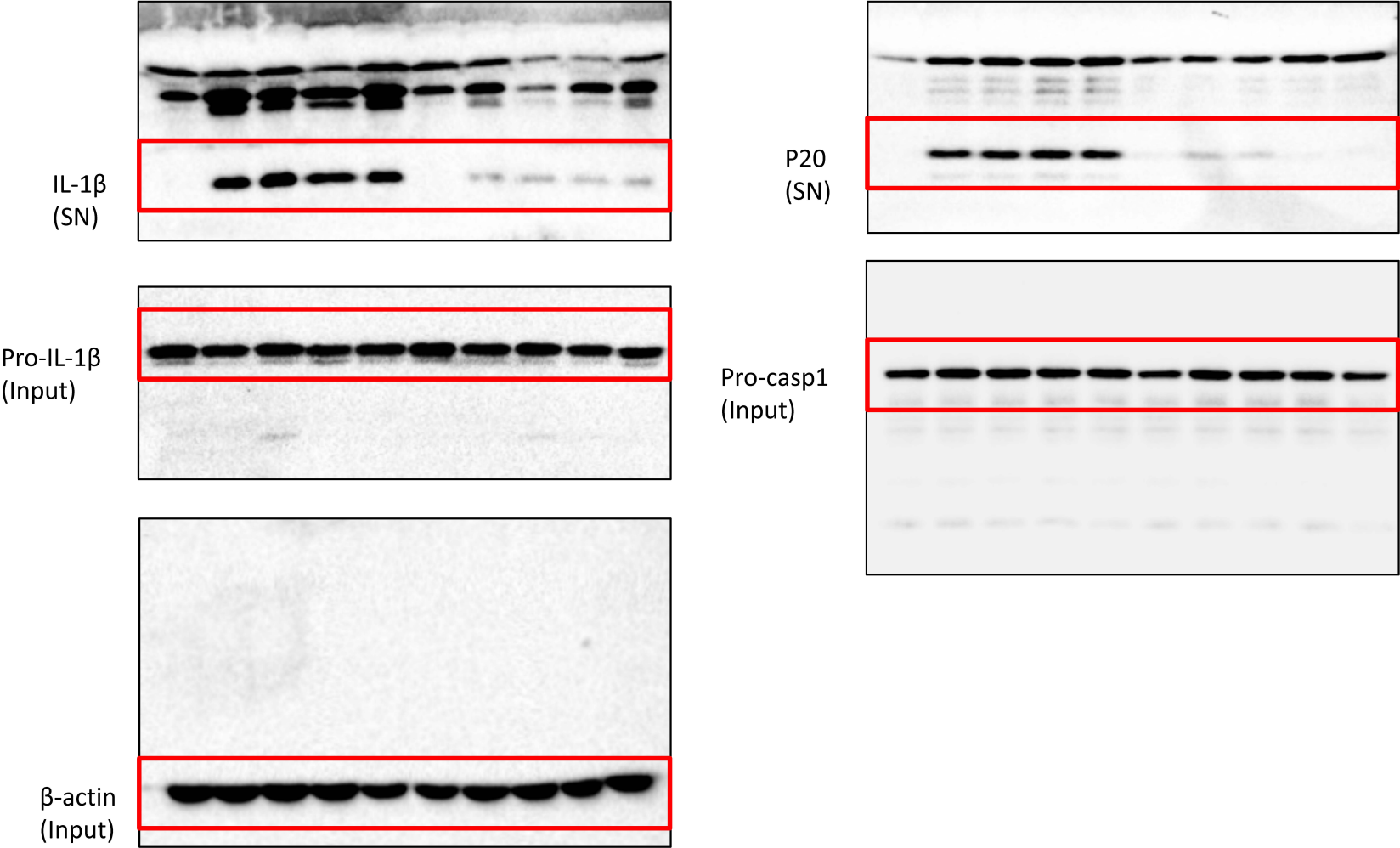

Figure 1H

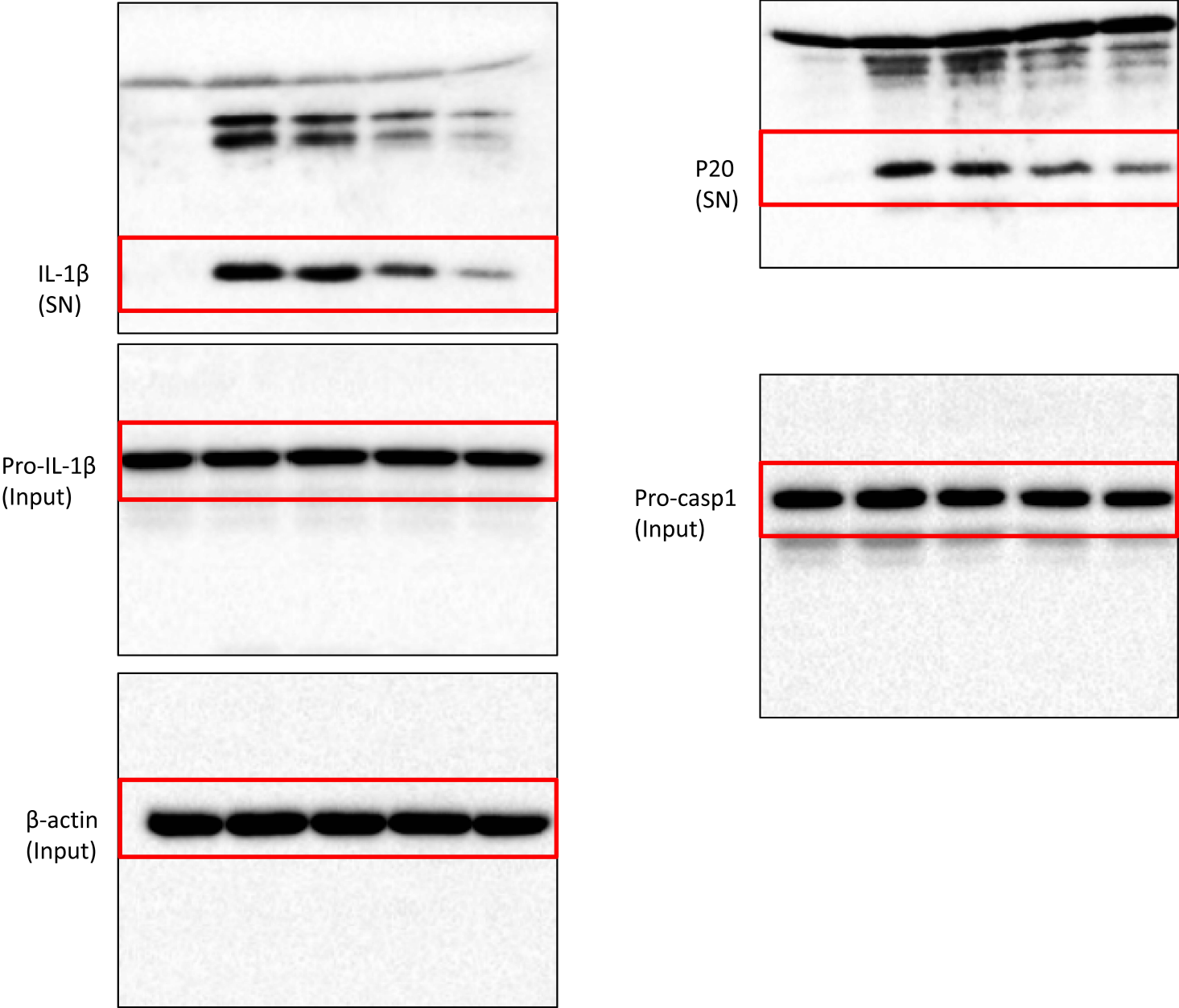

Supplement: Supplementary file 4 — Source Data for Figure 1 [file EMMM-10-e8689-s002.pdf]

Figure 2A

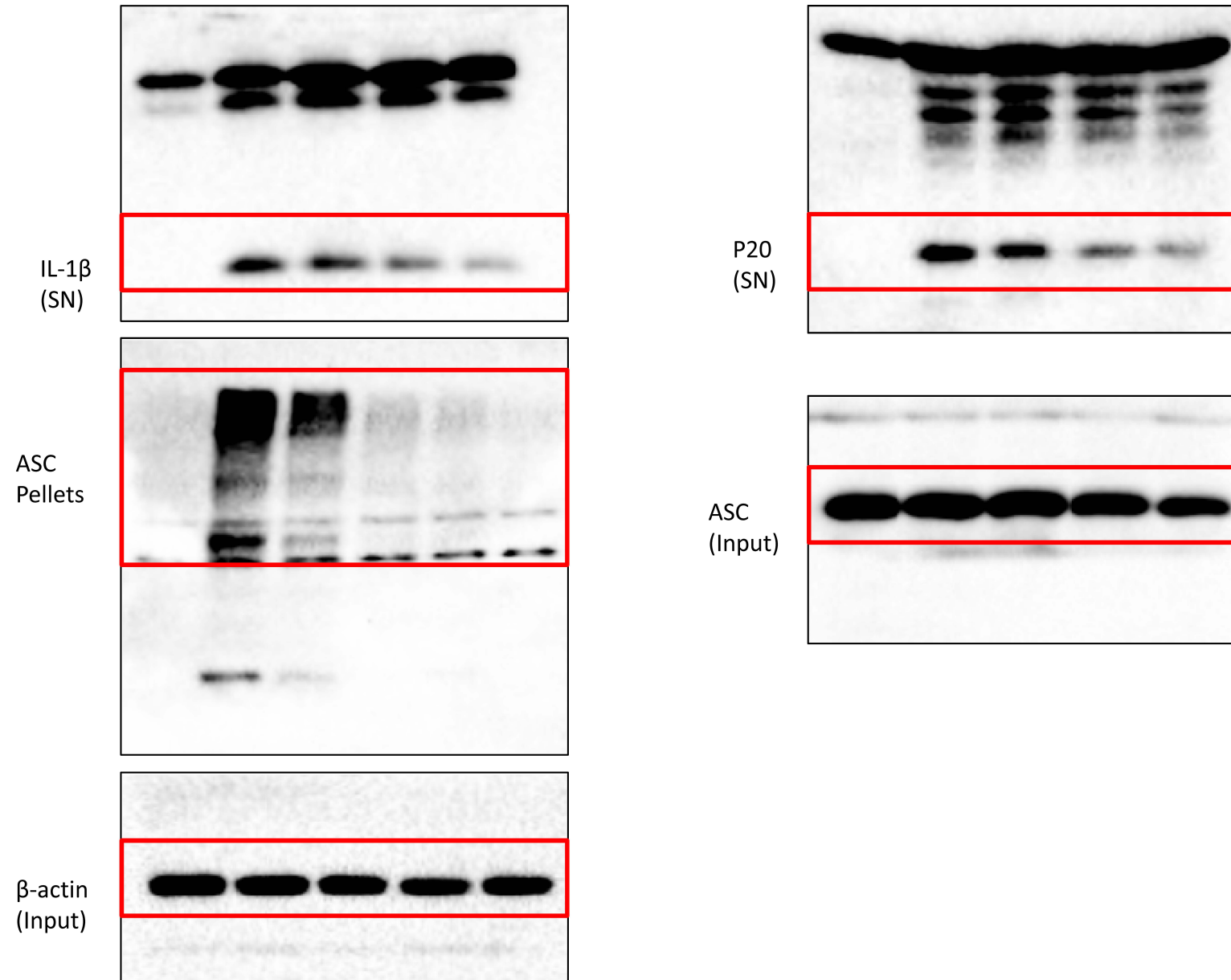

Figure 2B

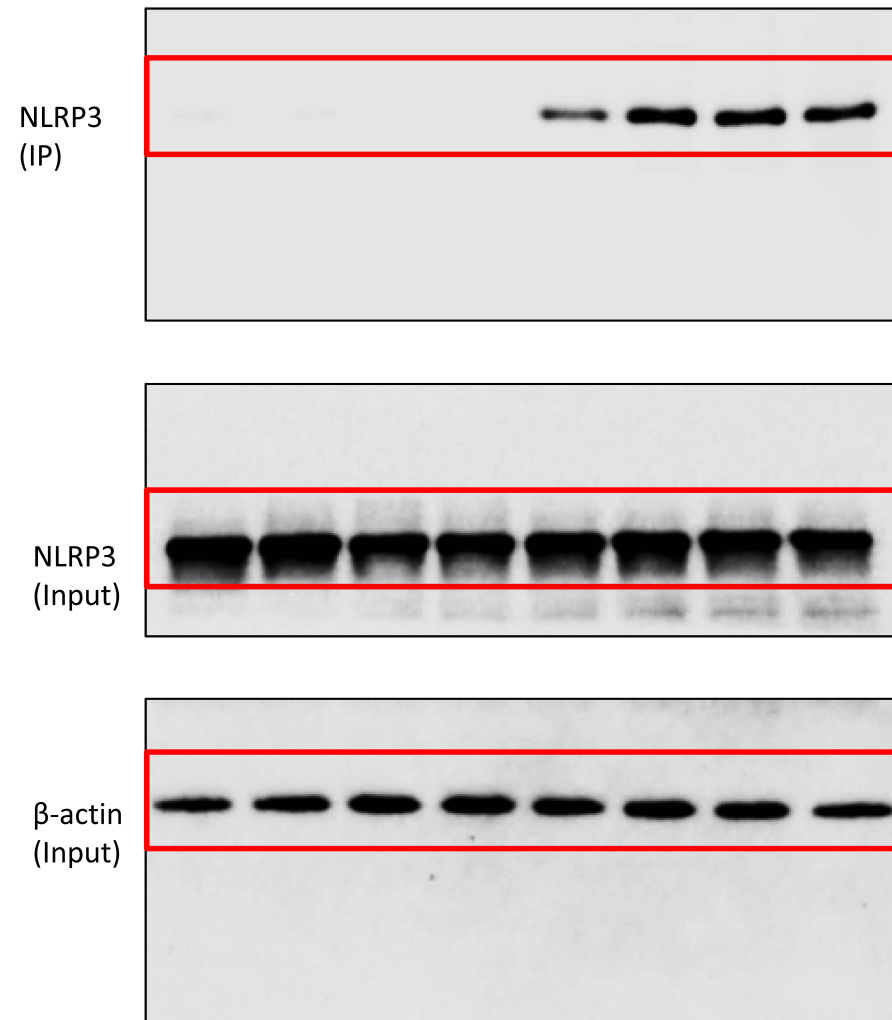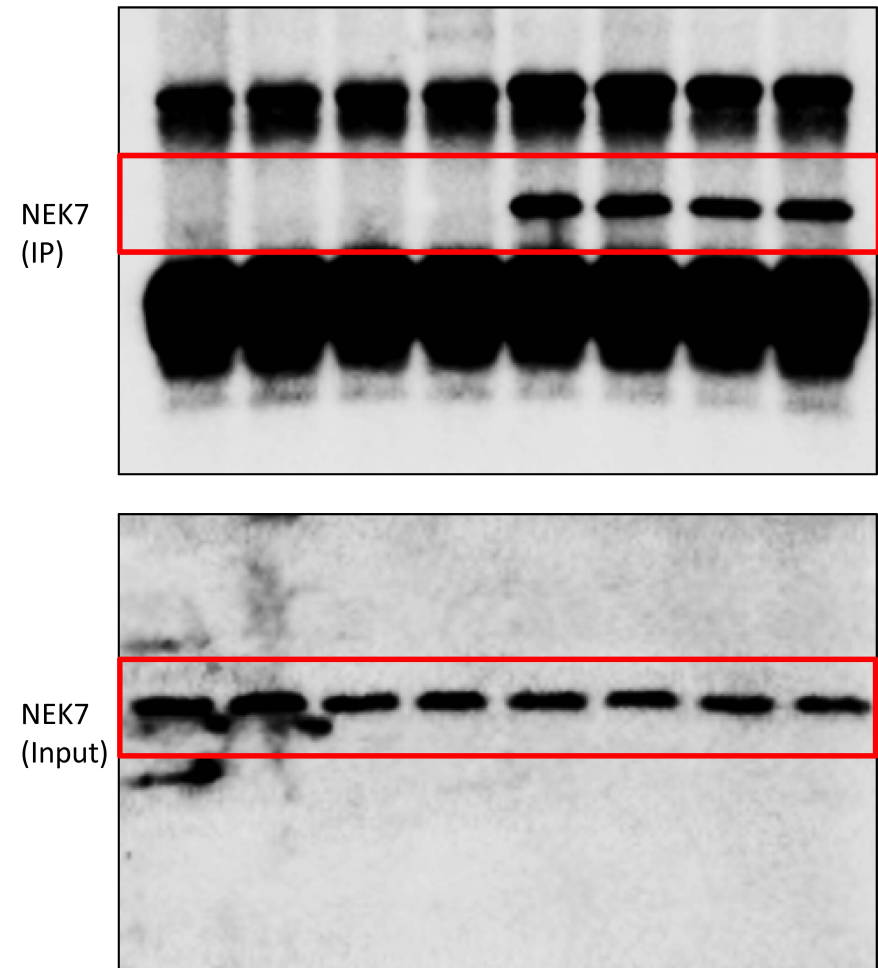

Figure 2C

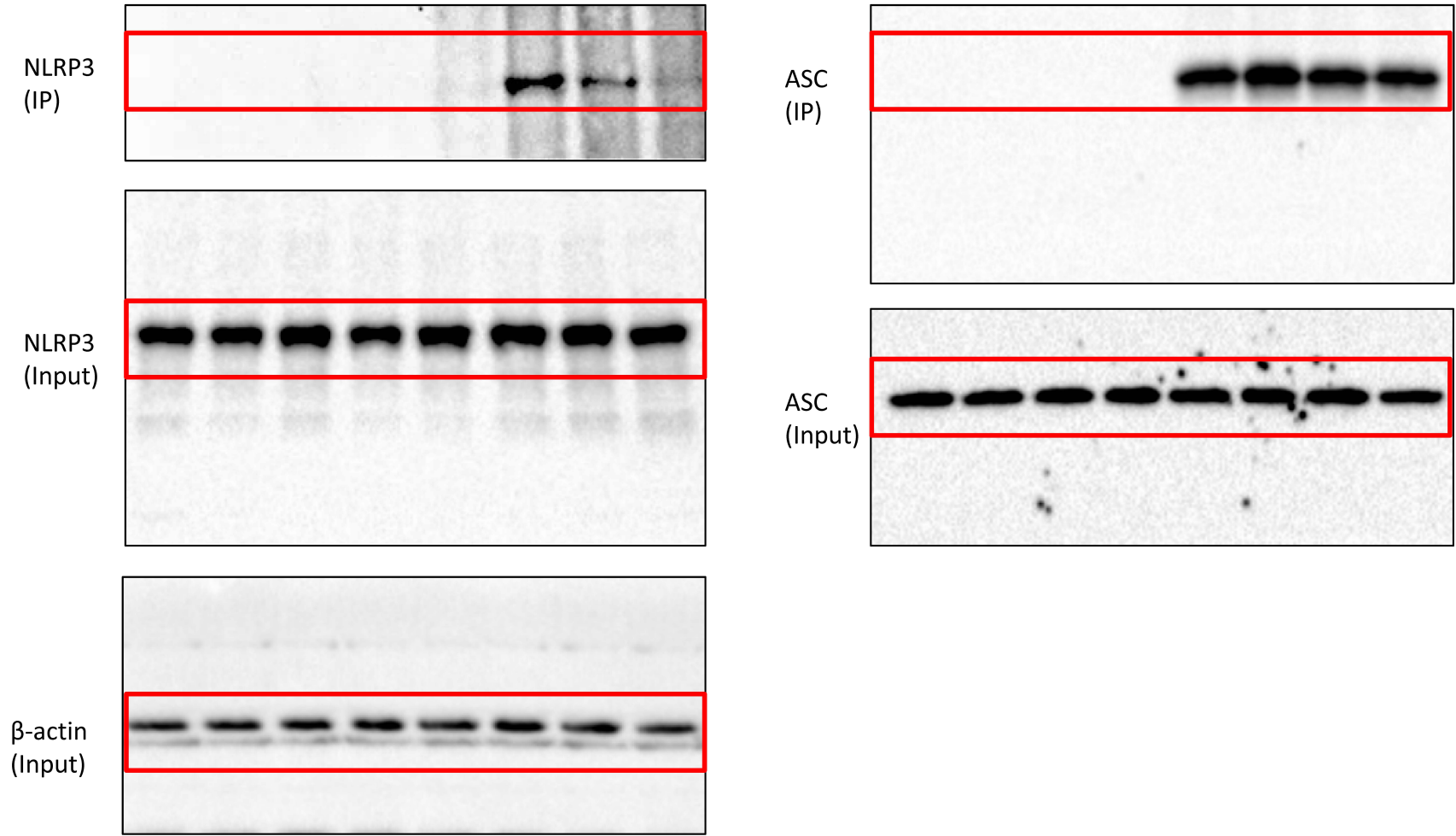

Supplement: Supplementary file 5 — Source Data for Figure 2 [file EMMM-10-e8689-s003.pdf]

Figure 3A

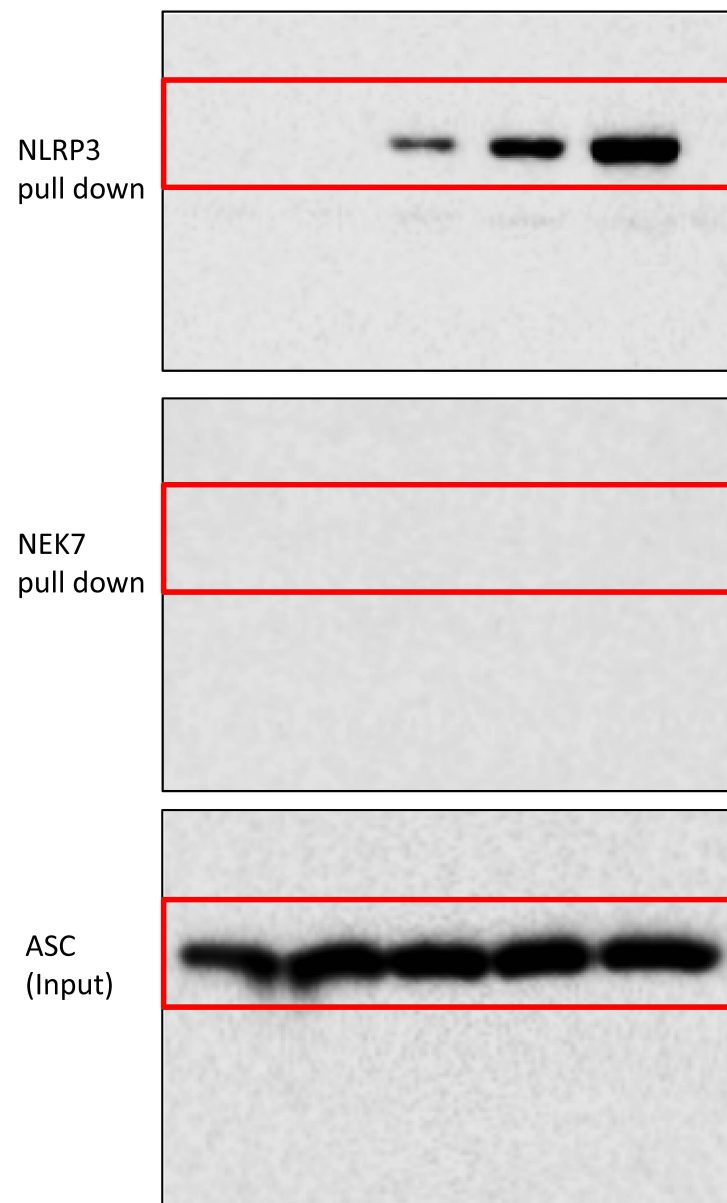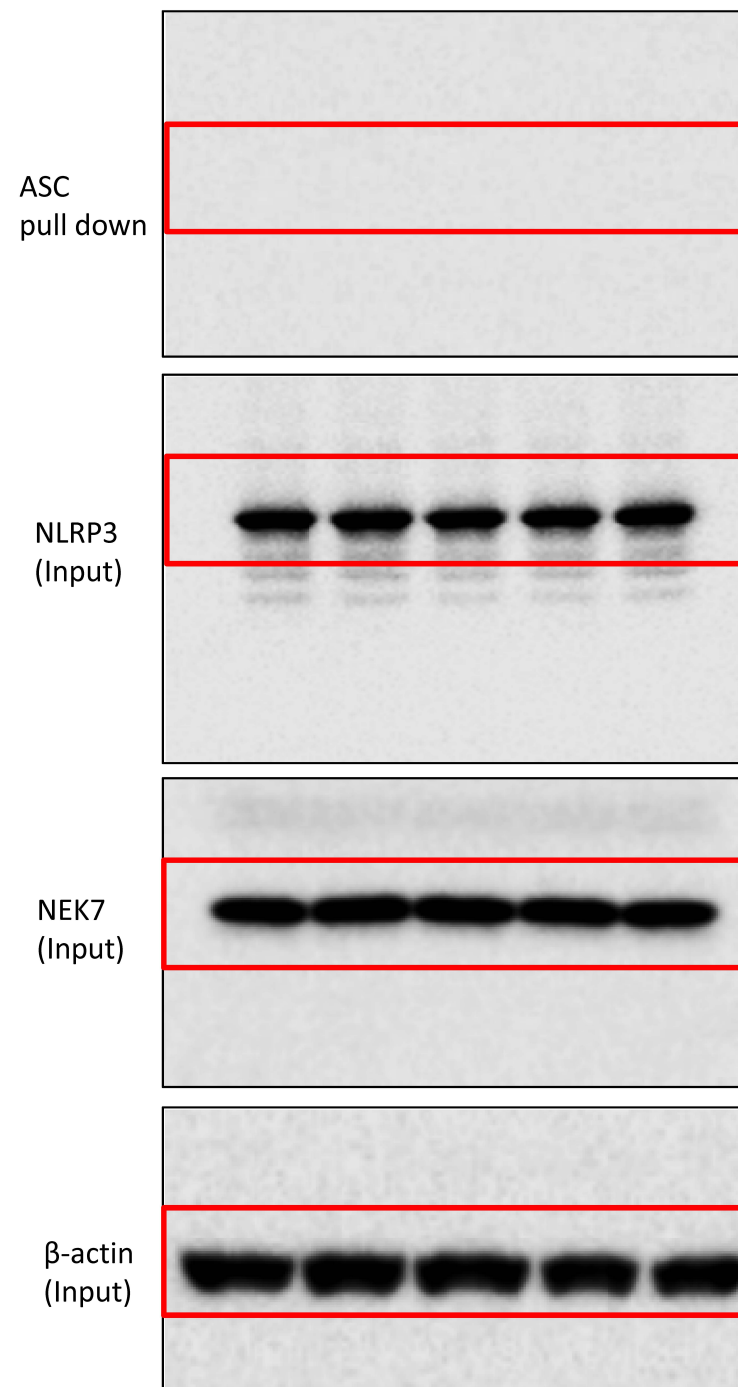

Figure 3B

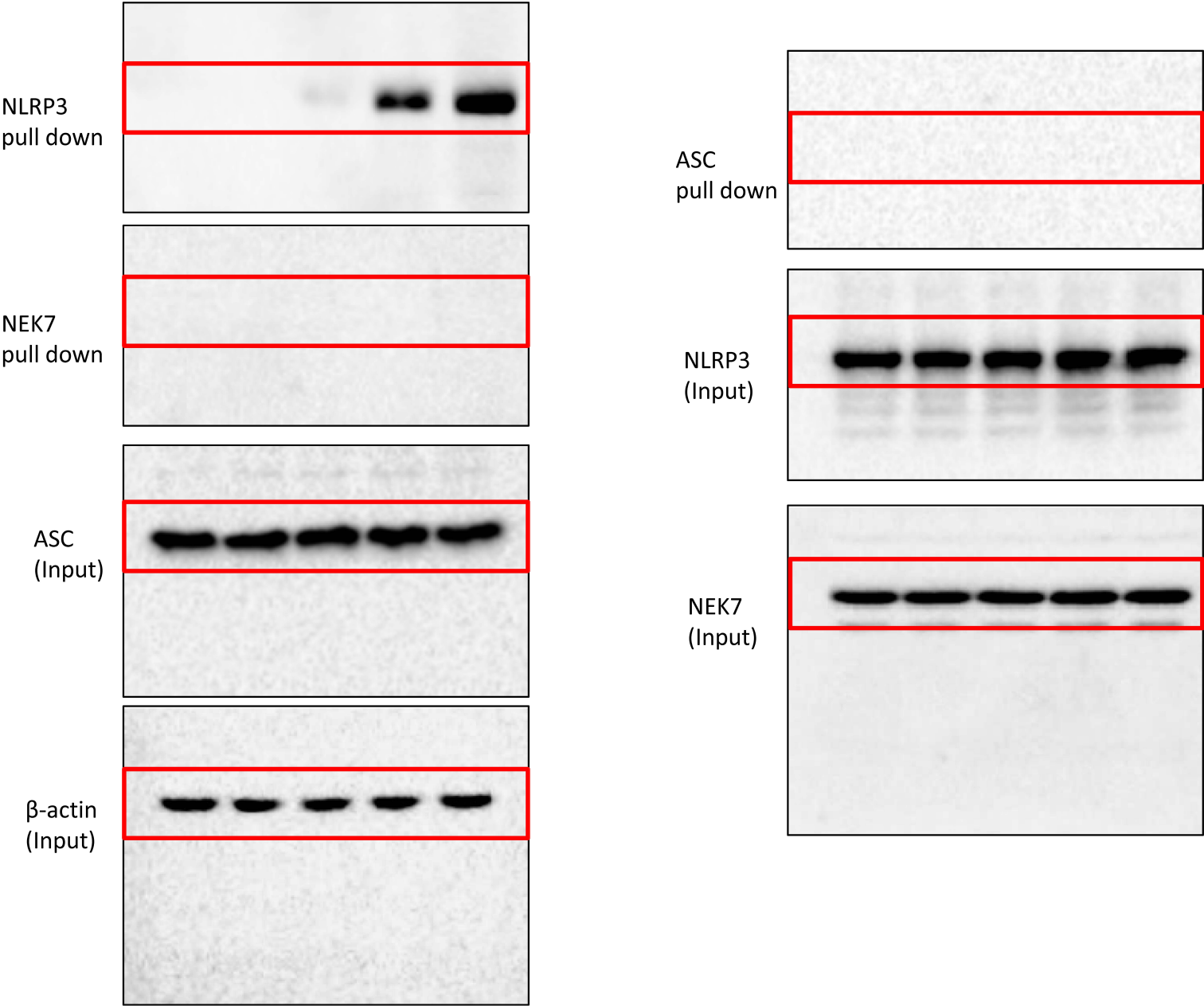

Figure 3C

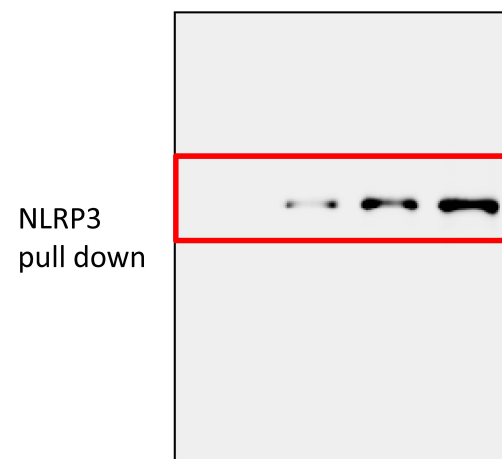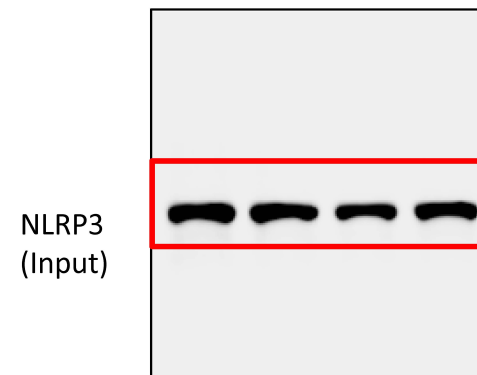

Figure 3D

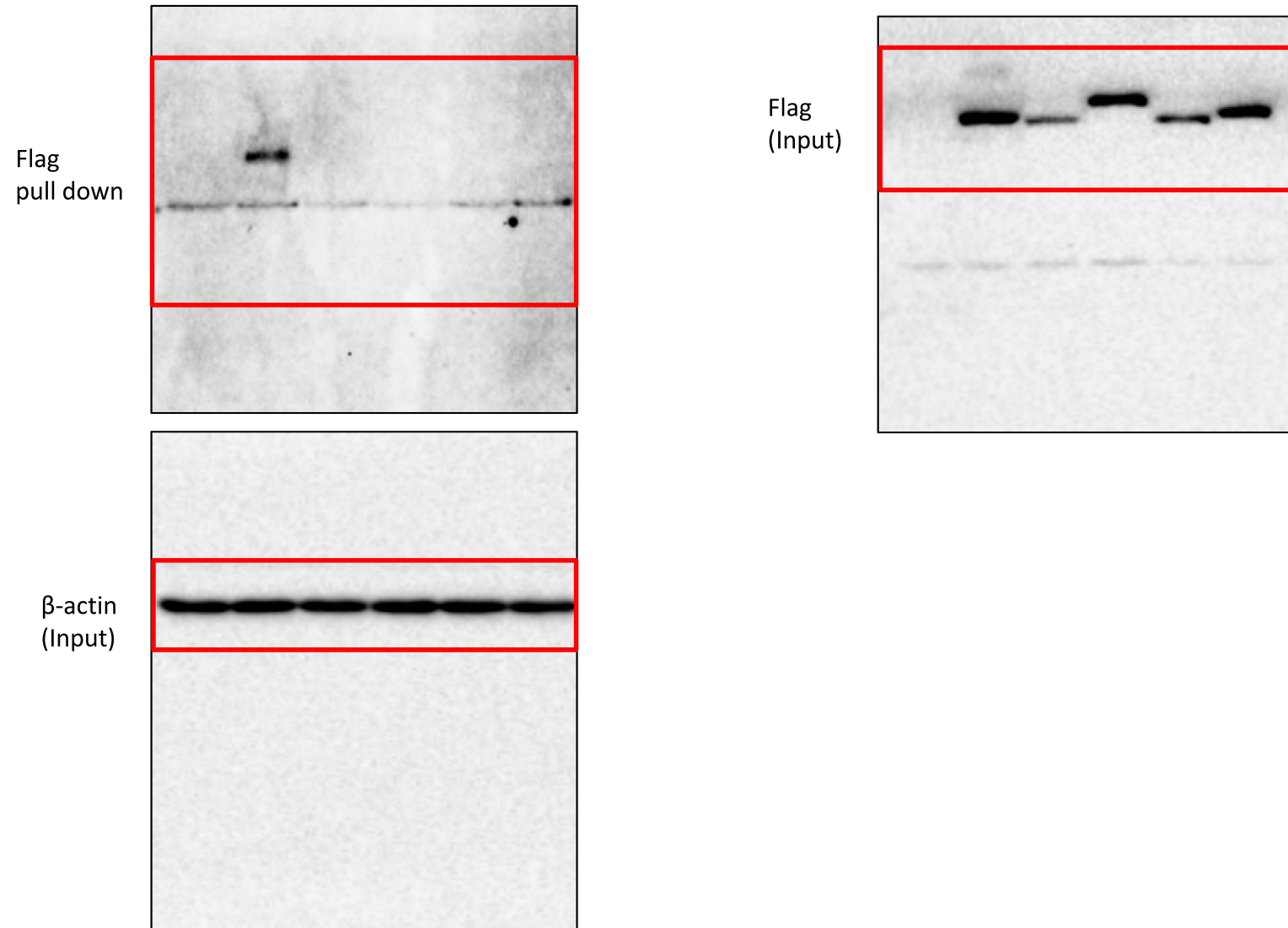

Figure 3E

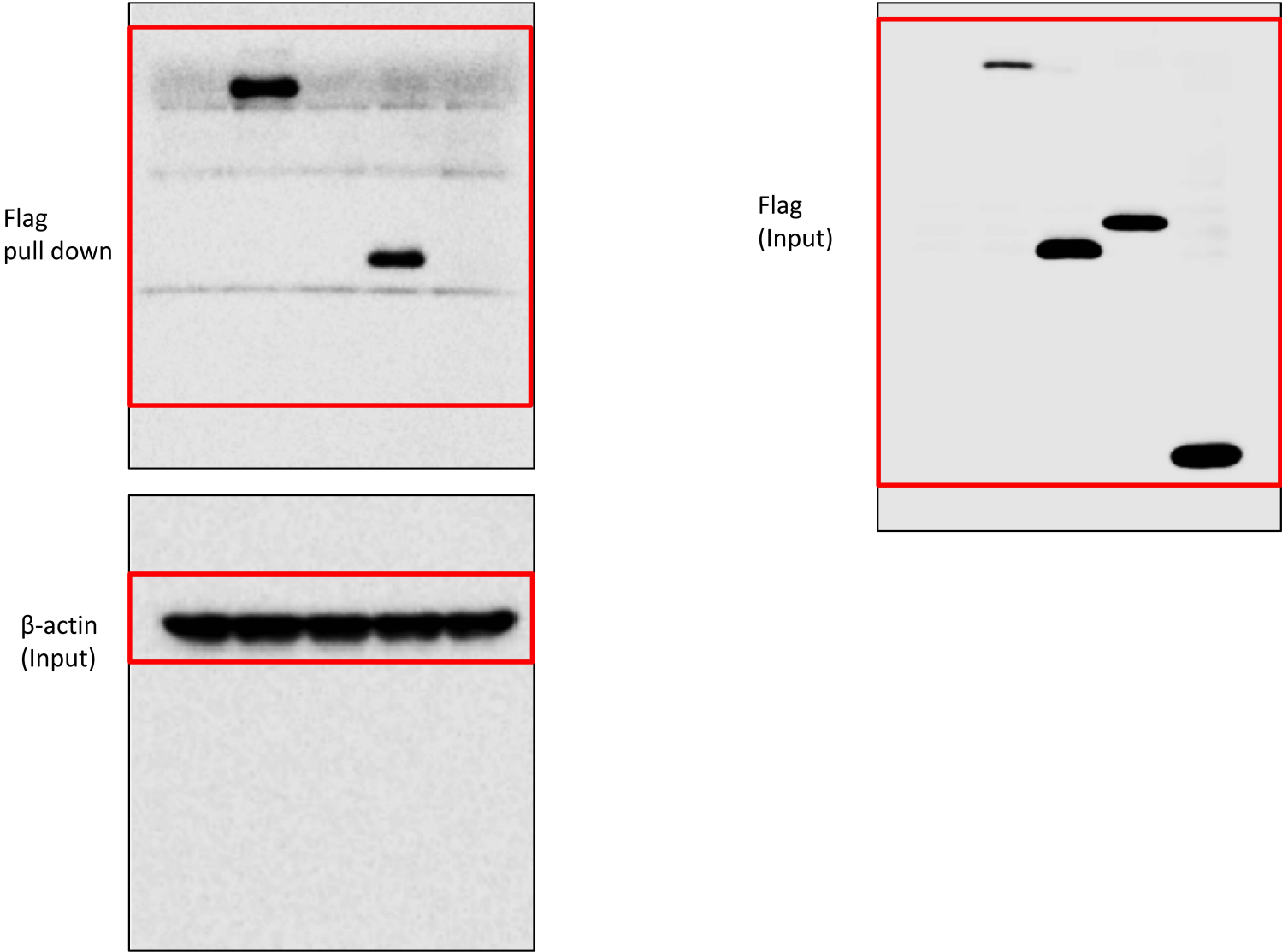

Figure 3F

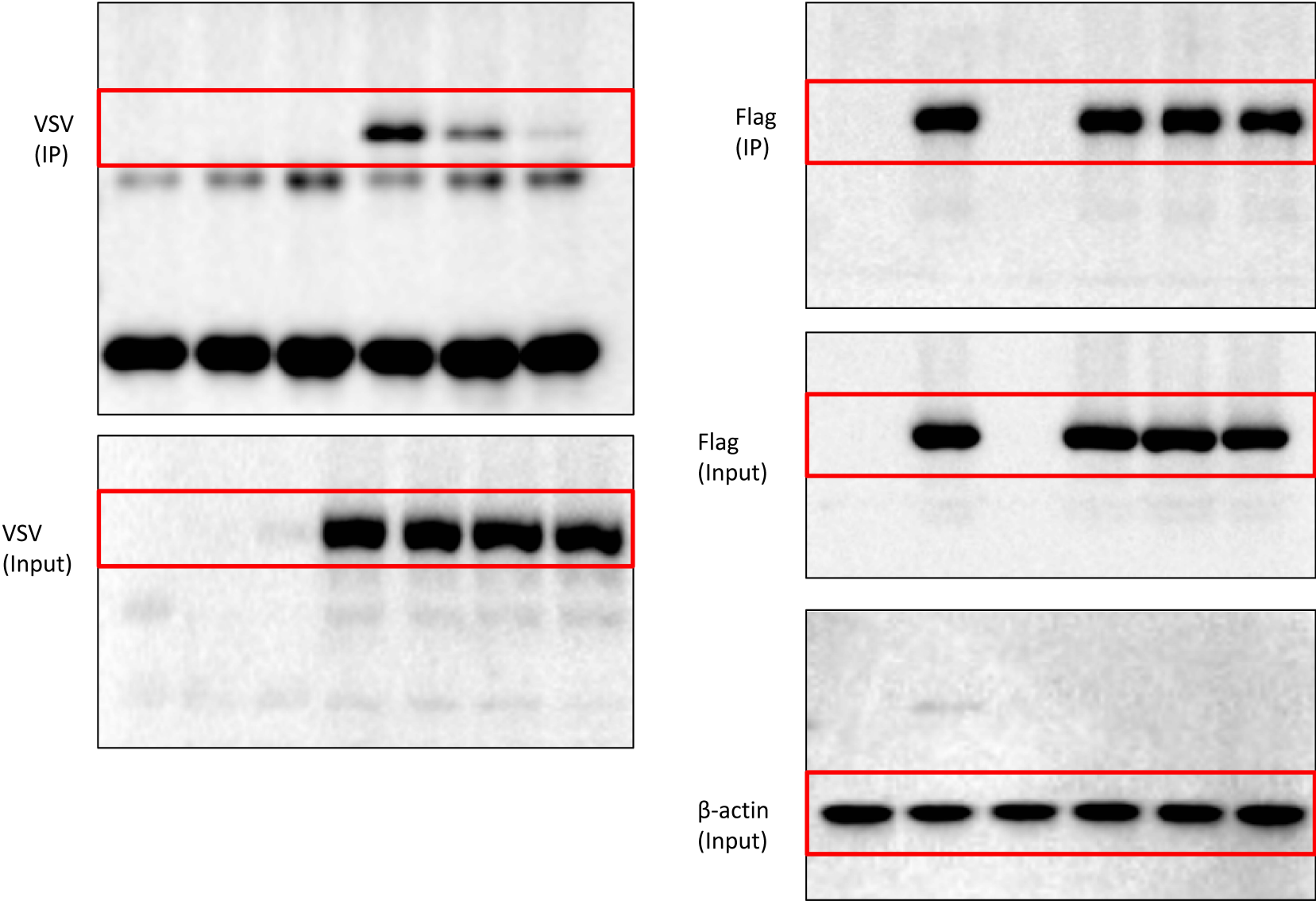

Figure 3G

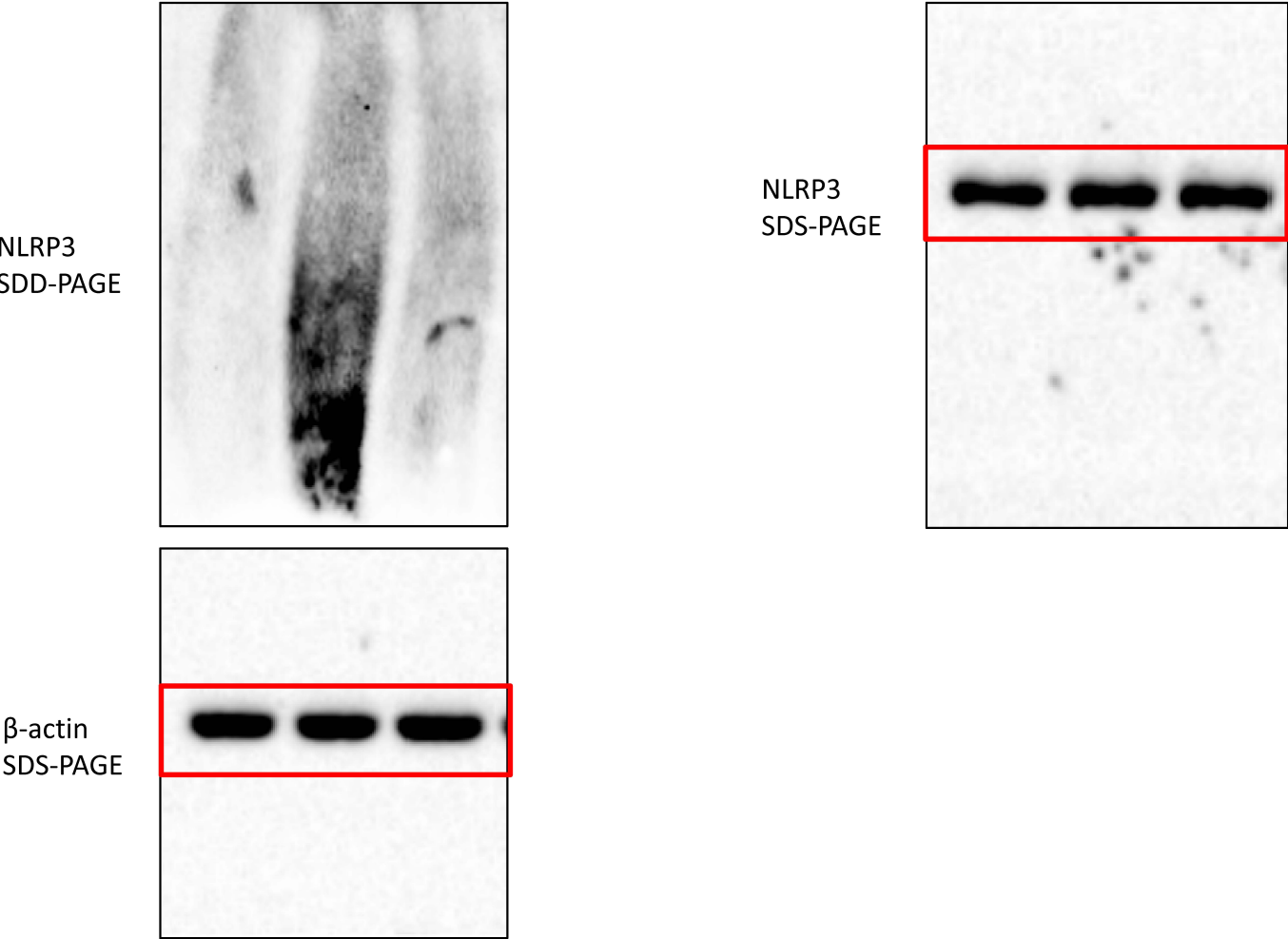

Supplement: Supplementary file 6 — Source Data for Figure 3 [file EMMM-10-e8689-s004.pdf]

Figure 5H

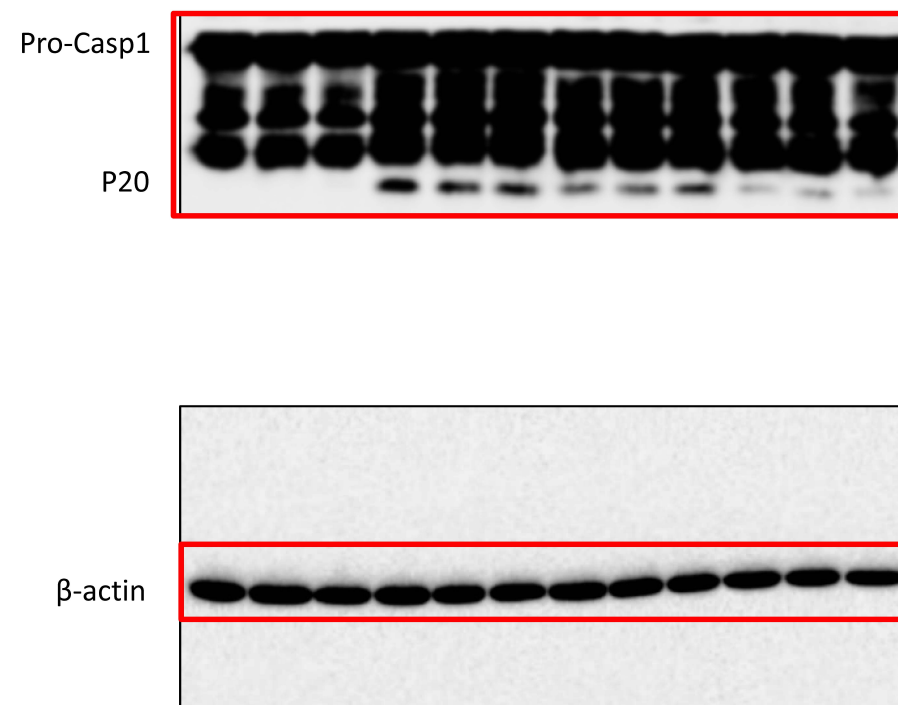

Supplement: Supplementary file 7 — Source Data for Figure 5 [file EMMM-10-e8689-s005.pdf]

Figure 7A

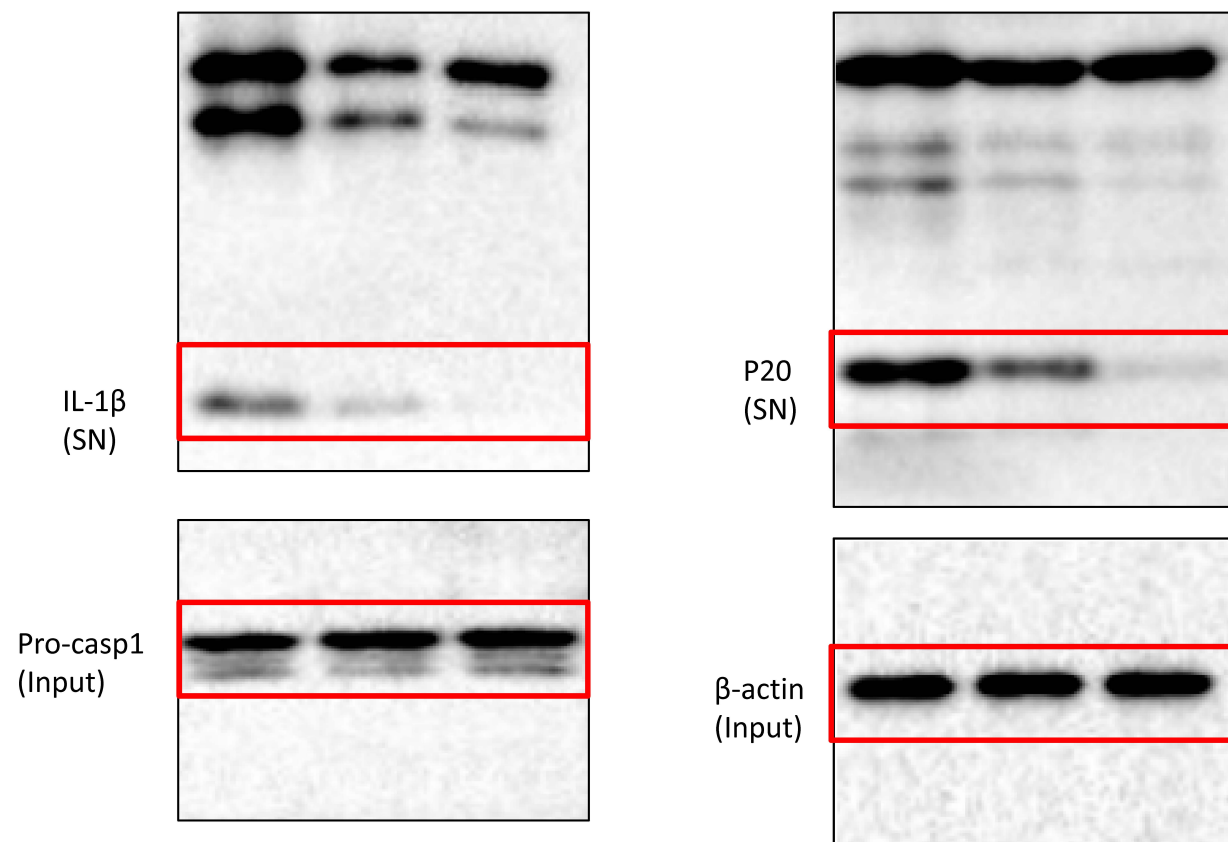

Supplement: Supplementary file 8 — Source Data for Figure 7 [file EMMM-10-e8689-s006.pdf]
